# Deciphering the Mutational Background in Citrin Deficiency Through a Nationwide Study in Japan and Literature Review

**DOI:** 10.1155/humu/9326326

**Published:** 2025-04-22

**Authors:** Jun Kido, Keishin Sugawara, Sotiria Tavoulari, Georgios Makris, Véronique Rüfenacht, Kimitoshi Nakamura, Edmund R. S. Kunji, Johannes Häberle

**Affiliations:** ^1^University Children's Hospital Zurich and Children's Research Centre, University of Zurich, Zurich, Switzerland; ^2^Department of Pediatrics, Kumamoto University Hospital, Kumamoto, Japan; ^3^Department of Pediatrics, Faculty of Life Sciences, Kumamoto University, Kumamoto, Japan; ^4^Medical Research Council Mitochondrial Biology Unit, University of Cambridge, Cambridge, UK

**Keywords:** citrin deficiency, mitochondrial aspartate/glutamate carrier, mitochondrial disease, SLC25 mitochondrial carrier family, urea cycle disorders

## Abstract

Citrin deficiency (CD) is an autosomal recessive disorder caused by the absence or dysfunction of the mitochondrial transporter citrin, resulting from mutations in *SLC25A13*. The disease presents with age-dependent clinical manifestations: neonatal intrahepatic cholestasis caused by CD (NICCD), failure to thrive and dyslipidemia by CD (FTTDCD), and an adult-onset form (formerly called Type II citrullinemia, CTLN2, recently renamed to “adolescent and adult citrin deficiency,” AACD). We performed this study to compile known genotypes found in CD patients and investigate their impact on the clinical course. Through a nationwide survey in Japan as well as a literature review, we collected information regarding 68 genetic variants of a total of 345 patients with CD (285 NICCD, 19 post-NICCD, and 41 AACD). In this cohort, the pathogenic variants, arising from nonsense, insertion/deletion, and splice site mutations, are expected to have severe functional or biogenesis defects. Of 82 alleles in patients with AACD, the two most common variants, c.852_855del and c.1177+1G>A, accounted for 25 alleles (30.5%) and 15 alleles (18.3%), respectively. The c.852_855del variant, even when present as part of compound heterozygosity, often presented with hyperammonemia (≥ 180 μmol/L), cognitive impairment, short stature (< -2SD), liver cirrhosis, and pancreatitis, with some patients requiring liver transplantation. In conclusion, certain *SLC25A13* genotypes are particularly frequent, especially those that result in severely truncated citrin proteins with often a significant impact on the clinical outcome of the patient. The most prevalent variant is c.852_855del, which was found in 42% (128/304) of NICCD/post-NICCD cases and 49% (20/41) of AACD patients.

## 1. Introduction

Citrin is a calcium-binding mitochondrial aspartate/glutamate carrier located in the mitochondrial inner membrane and is highly expressed in the liver mitochondria as well as in many other organs, including the kidney, pancreas, and heart. It is encoded by the *SLC25A13* gene on chromosome 7q21.3, which contains 18 coding exons and encompasses almost 201 kb of DNA, encoding a 675-amino acid protein with a molecular mass of 74 kDa [1].

Citrin deficiency (CD) is an autosomal recessive disorder caused by pathogenic mutations of the *SLC25A13* gene [1] and presents with age-dependent clinical manifestations [2–4]: neonatal intrahepatic cholestasis by CD (NICCD: OMIM #605814), failure to thrive and dyslipidemia by CD (FTTDCD), and an adult form known as adult-onset Type II citrullinemia (CTLN2, OMIM #603471), which has recently received the new terminology “adolescent and adult citrin deficiency” (AACD) [5] to avoid potentially tragic confusion with classical citrullinemia Type I [6]. Saheki et al. first reported CD in adult patients as a condition characterized by decreased hepatic argininosuccinate synthetase 1 (ASS1) activity with normal kinetic properties and heat stability [7, 8]. Later, Kobayashi et al. from the same research group suggested that the primary cause of this condition was not derived from the *ASS1* gene locus [9], succeeded in cloning the causative gene *SLC25A13* [1] and coined the term “citrin” as the relevant protein in CD. Finally, it was found that the gene *SLC25A13* encodes for the mitochondrial aspartate/glutamate carrier 2 (AGC2) [10], which imports glutamate with a proton and exports aspartate [11]. Citrin and the related aralar (SLC25A12) [10] are exceptional members of the SLC25 mitochondrial carrier family, the largest transporter family in humans [12]. They exist as homodimers and each protomer has a three-domain architecture, comprising of a calcium-binding N-terminal domain with eight EF-hands, a SLC25 mitochondrial carrier domain responsible for substrate transport and a C-terminal domain, comprising of an amphipathic helix [13].

CD can manifest itself with a diverse set of symptoms in different stages of life. Neonates or infants with CD often develop intrahepatic cholestasis and diverse metabolic abnormalities, including citrullinemia, galactosemia, hypoglycemia, and hyperammonemia [14]. This disease presentation is regarded as NICCD, and the clinical manifestations of NICCD often resolve spontaneously at the end of infancy even without intervention. In the post-NICCD period, some patients present with failure to thrive, dyslipidemia, recurrent hypoglycemia, and frequent symptoms of fatigue, a condition classified as FTTDCD [3]. Most of these patients prefer protein- and lipid-rich foods, such as fried chicken, and a self-select carbohydrate-restricted diet. Moreover, a silent remission period with apparently no symptoms may continue even after adolescence. However, up to 20% of patients with CD may suddenly develop a life-threatening metabolic disease, AACD (formerly CTLN2), which is characterized by severe liver steatosis accompanied by hyperammonemia, cognitive impairment, and sudden episodes of unconsciousness due to brain edema [15, 16].

CD is a more prevalent condition in East Asia, particularly in Japan and China [17]. The incidence is 1 in 17,000 births in Asia [18] with a carrier frequency of *SLC25A13* variants estimated to be 1/47–1/67 in China [18–20], 1/57 in Taiwan [18], 1/31 in Vietnam [21], 1/41 in Singapore [22], 1/90 in Thailand [23], 1/69–1/74 in Japan [18, 24], and 1/112 in Korea [18]. There are increasing numbers of reports of CD from Western countries and other parts of the world, where the frequency is lower [16, 25–32]. There are several reports describing *SLC25A13* variants in patients with CD, of which about 11 mutations were reported especially prevalent in Japanese patients [18, 24, 33–35]. While the clinical course in patients with CD is already well documented in the literature, there is a lack of combined clinical and genetic information in this condition. CD is exceptional in both the number of different mutations as well as the frequency of their occurrence compared to all other mitochondrial diseases associated with SLC25 mitochondrial carrier family [12].

To combine clinical and genetic information for this disease thoroughly, we made use of our previous nationwide study in Japan, in which clinical manifestations, dietary and medical interventions, and the long-term outcomes in Japanese patients with CD had been reported [36]. We compared those clinical data in the present study with the corresponding genotypes from this patient cohort. To a total of 187 patients from the nationwide study, we added data from patients reported in the literature and performed a combined analysis. This analysis allowed us to obtain an unprecedented insight into the mutational background of CD combined with the corresponding clinical manifestations.

## 2. Material and Methods

### 2.1. Patient Cohorts

Previously, we had conducted a nationwide survey on Japanese patients with CD [36]. In this survey, we acquired the clinical data of 222 patients (192 NICCD, 91 males and 101 females; 13 post-NICCD, 6 males and 7 females; and 17 AACD, 11 males and 6 females). These patients were diagnosed and/or treated in different departments, including pediatrics, neonatology, endocrinology and metabolism, genetics, and transplant surgery from 104 institutions between January 2000 and December 2019. In addition to this study, we collected data of CD patients diagnosed in a single institution in Kumamoto since 2020. After excluding patients with incomplete information, we analyzed clinical and genetic data from 187 patients with CD (174 NICCD/FTTDCD and 13 AACD), of which 38 patients (all NICCD) were sibling cases. As described, 18 patients within this cohort had a pathogenic variant detected in only a single allele [36].

### 2.2. Patient Literature Review

As part of the literature review, we searched for the genetic and clinical information of patients with CD available in PubMed (https://pubmed.ncbi.nlm.nih.gov) or Google Scholar (https://scholar.google.com) using the keywords “citrin deficiency,” “*SLC25A13* variant,” and “mutation” (access date on 5 October 2023). From this, we included 196 patients with CD reported in 63 papers. If patients from any of those reports had already been included in the nationwide study (as identified through data comparison and individual verification with colleagues), the information from the nationwide study was used. This was the case for 38 patients, and after excluding those, we were able to include 130 patients from the literature, of which nine patients (seven NICCD and two AACD) were sibling cases. Of these 130 patients, 28 patients had a pathogenic variant only in a single allele with no pathogenic variant detected in the other allele. Importantly, there was little variation in the methods used for detection of *SLC25A13* variants, since centers used single-gene Sanger sequencing, following a published protocol [18, 33, 37], while no whole exome or genome sequencing was performed.

### 2.3. Variant Nomenclature and Severity Prediction

Variant nomenclature used in this study follows the guidelines established by the Human Genome Variation Society (http://varnomen.hgvs.org/) [38], and all variants were listed including their protein level descriptions. The public database ClinVar (http://www.ncbi.nlm.nih.gov/clinvar) [39] and the bioinformatics tools PolyPhen-2 (http://genetics.bwh.harvard.edu/pph2) [40] and SIFT (https://sift.bii.a-star.edu.sg/) [41] were used for adding the predicted impact of sequence variants on the function of the citrin transporter.

### 2.4. Statistical Analysis

The residual analysis to compare the number of variants of c.852_855del (p.Met285Profs∗2), c.1177+1G>A (p.Val340_Arg392del), IVS16ins3kb, c.1311+1G>A, c.674C>A (p.Ser225∗) (p.Val411_Cys437del), c.1638_1660dup (p.Ala554Glyfs∗17), and c.1478A>G (p.Asp493Gly) between NICCD and AACD was performed using IBM SPSS Statistics Version 25. A *p*-value of < 0.05 was considered statistically significant.

## 3. Results

We collected information regarding 68 *SLC25A13* variants in a total of 345 patients with CD (285 NICCD, 19 post-NICCD, and 41 AACD), who were identified through a nationwide study in Japan or through literature review. A complete list of all *SLC25A13* variants and related information is provided in Data S1. Out of the entire cohort of 345 patients, biallelic variants were found in 301 patients, while 44 patients exhibited only a single defective allele.

In a total of 304 NICCD patients (153 males, 132 females, and 19 with unknown gender), we identified 562 affected alleles (Table 1). Of these 304 patients, 194 were Japanese [36, 42–47], and the other 110 were reported from either Chinese [3, 21, 48–61], Taiwanese [62–65], Korean [66, 67], Malaysian [68–70], Pakistani [25, 28], Turkish [71, 72], European [16, 25–32], or Asian origin [16, 73].

The median age of onset in this NICCD cohort was 1 month (IQR: 1–3 months). Median age at diagnosis was 3 months (IQR: 1–5 months) though this information was available for only 183 Japanese patients. Of 562 alleles in patients with NICCD or post-NICCD, the variant c.852_855del was found in 157 alleles (27.9%), and c.1177+1G>A was found in 143 alleles (25.4%) (Table 1).

**Figure 1:**
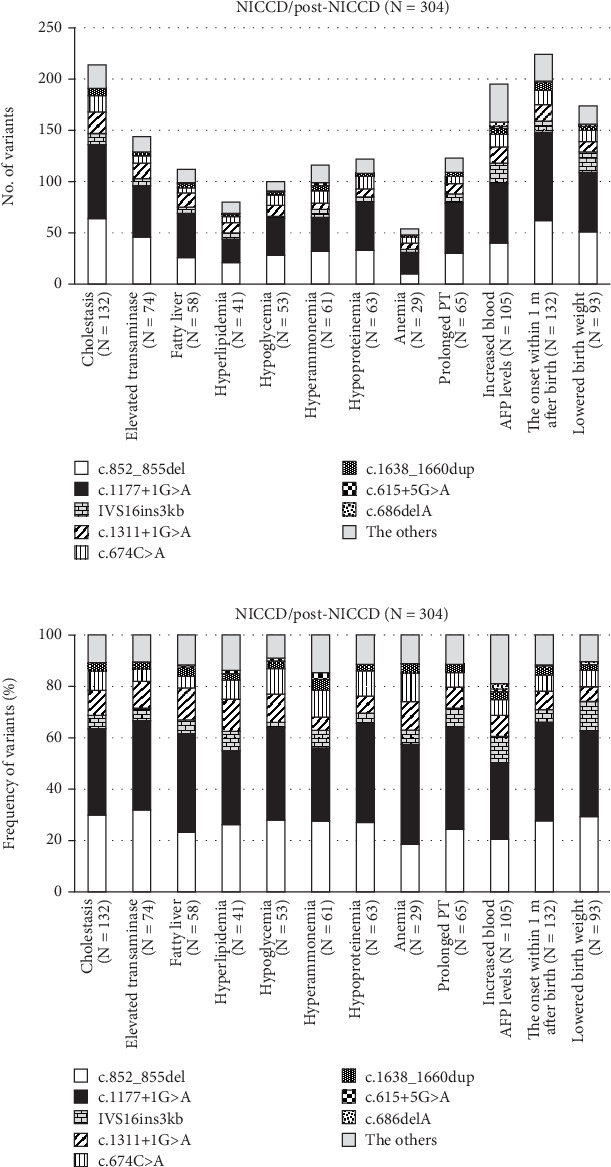
The most common clinical manifestations and variants in *SLC25A13* in 285 patients with NICCD and 19 patients with post-NICCD. The frequency of variants (a) and relative frequency of variants (b) of *SLC25A13* in the most common clinical manifestations in NICCD.

**Figure 2:**
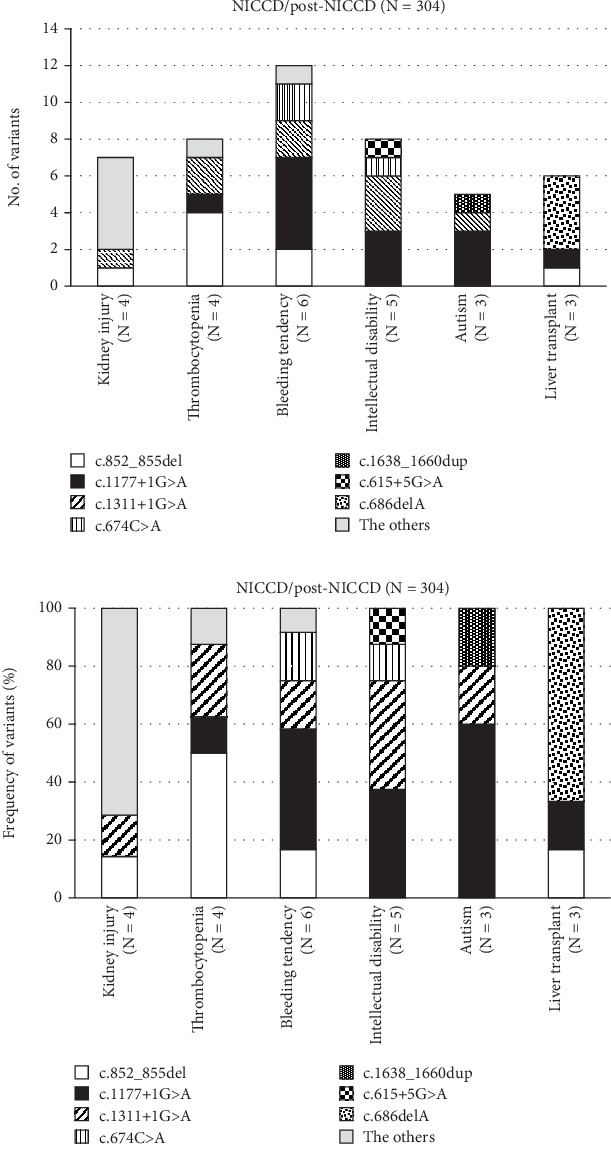
The less common clinical manifestations and variants in *SLC25A13* in in 285 patients with NICCD and 19 patients with post-NICCD. The frequency of variants (a) and relative frequency of variants (b) of *SLC25A13* in less common clinical manifestations in NICCD/post-NICCD.

An even higher proportion of these most frequent variants was observed in 132 NICCD patients affected by cholestasis, in 74 patients affected by elevated transaminases (≥ 100 U/L), and in 58 patients affected by fatty liver, in which the c.852_855del variant was present on 68 alleles (29.9%, 64/214), 65 alleles (31.9%, 46/144), and 26 alleles (23.2%, 26/112), respectively (Figures 1 and 2). Moreover, the c.1177+1G>A variant was present on 105 alleles (33.6%, 72/214), 94 alleles (34.7%, 50/144), and 43 alleles (38.4%, 43/112), respectively.

Details to the clinical situation of NICCD and specific *SLC25A13* variants are compiled in Data S2 and S3. While some alleles, specifically c.852_855del and c.1177+1G>A, are often found in severely affected patients, there is an incomplete penetrance as some patients with those same alleles do not develop cholestasis, elevated transaminases, or fatty liver. In detail, 8, 14, and 36 patients carrying the c.852_855del variant did not show cholestasis, elevated transaminases, or fatty liver, respectively (Data S4). In the noncholestasis group, one patient was homozygous for c.852_855del, and seven patients were compound heterozygous for c.852_855del plus another variant (details provided in Data S4).

**Figure 3:**
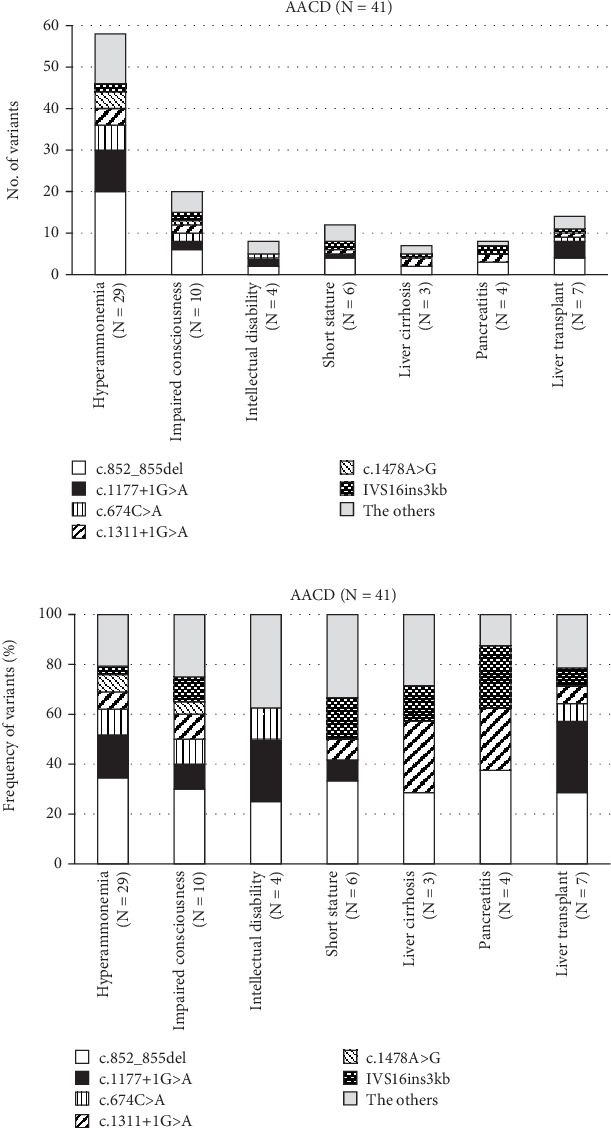
The phenotype and variants in *SLC25A13* in 41 patients with AACD. The frequency of variants (a) and relative frequency of variants (b) of *SLC25A13* and clinical manifestations in AACD. Homozygosity for c.852_855del was present in five patients with hyperammonemia, one patient with impaired consciousness, one patient with intellectual disability, one patient with short stature, one patient with liver cirrhosis, and one patient with pancreatitis. Homozygosity for c.1177+1G>A was present in two patients with hyperammonemia, one patient with intellectual disability, and one patient with liver transplantation.

In patients with AACD (formerly CTLN2), the most severe form of CD, we identified 82 affected alleles in a total of 41 patients (Table 2). Of these 41 AACD patients (28 males and 13 females), 31 were Japanese [36, 74–84], and 10 were either French-Canadian [25], Indian [85, 86], Chinese [87–89], Taiwanese [90], Turkish [71], Pakistani [91], or of other origin [92]. The median age of onset in this subgroup was 42 years (IQR: 25–60 years). The median age at diagnosis was 50 years and 9 months (IQR: 33–61 years and 1 month) though this parameter could only be evaluated in 13 Japanese patients. Of those 13 patients, 10 were reported to have specific eating habits, such as preference of fat-rich food and/or aversion to sugar- and carbohydrate-rich foods. In the 41 patients with AACD, the variant c.852_855del accounted for 25 alleles (30.5%) and c.1177+1G>A for 15 alleles (18.3%) (Table 2 and Data S5). An even higher proportion for these most frequent variants was found in 29 AACD patients affected by hyperammonemia (≥ 180 μmol/L) and in 10 patients affected by impaired consciousness, in which the c.852_855del variant was present on 21 alleles (36.2%, 21/58) and 6 alleles (30.0%, 6/20), respectively, and the c.1177+1G>A variant was present on 11 alleles (19.0%, 11/58) and 2 alleles (10%, 2/20), respectively (Figure 3). Details to the clinical situation of AACD and specific *SLC25A13* variants are compiled in Data S5. Although only three patients developed liver cirrhosis and/or pancreatitis, the variants c.852_855del, c.1018+1G>A, c.1311+1G>A, and IVS16ins3kb were present in the patients affected by those complications (Figure 3 and Data S5).

The frequency of specific variants in AACD compared to NICCD was higher for c.852_855del (25/157) than for c.1177+1G>A (15/143), although this difference was not significant (*p* = 0.083). Likewise, the frequency of other variants (IVS16ins3kb (4/47), c.674C>A (7/26), c.1311+1G>A (6/26), and c.1638_1660dup (0/23)) was not significantly different between patients with AACD and NICCD (all *p* > 0.05). Only the variant c.1478A>G was significantly more frequent (*p* < 0.001) in AACD than in NICCD (4/0), but these numbers are very small. Finally, also, the frequency of clinical manifestations in patients with AACD including hyperammonemia (21/11), impaired consciousness (6/2), intellectual disability (2/2), short stature (4/1), liver cirrhosis (2/0), pancreatitis (1/0), and liver transplant (4/2) between c.852_855del and c.1177+1G>A (25/15) was without significant difference (all *p* > 0.05) (Figure 3).

## 4. Discussion

The molecular basis of CD in patients could have only been described following the identification of *SLC25A13* as the causative gene for this condition [1, 10, 93]. Accordingly, information about pathogenic mutations is primarily available as part of case reports or case series, but it is less systematically documented. So far, a total of 651 *SLC25A13* variants are registered in ClinVar database, of which 147 have been reported as pathogenic or likely pathogenic. Here, we aimed to provide a broader analysis of the molecular background of the disease by combining mutation data from a large nationwide study in Japan with a comprehensive collection of respective literature data containing clinical information.

The basis for the present study is the sequence variants in 304 NICCD patients and 41 AACD patients. While there appeared to be a relative preference for a few prevalent variants in AACD patients, for instance, c.852_855del being the most common variant in AACD and associated with a severe phenotype including hyperammonemia and impaired consciousness, a remarkable interpatient variability in NICCD patients with or without cholestasis and fatty liver, even carrying the same genotypes, was evident.

Variants c.1177+1G>A (34.3%; 112/326), c.852_855del (27.9%; 91/326), c.1311+1G>A (8.0%; 26/326), c.674C>A (5.5%; 18/326), IVS16ins3kb (4.3%; 14/326), and c.1638_1660dup (2.8%; 9/326) were overall the most prevalent in Japan [36] and the most prevalent five variants in AACD (Table 2). The c.852_855del variant has an allele frequency of > 90% in patients from Vietnam [94], around 60% in patients from China [95], and is found in almost 30% of alleles in patients from Japan and Korea [17, 24, 37, 96].

In the subgroup of 10 patients (three NICCD and seven AACD), who underwent liver transplantation, one NICCD patient was compound heterozygous for c.852_855del and c.1177+1G>A, and four AACD patients were compound heterozygous for c.852_855del (Data S3 and S5). The impact of c.852_855del on citrin function is expected to be deleterious due to the loss of both the carrier and C-terminal domains. However, other variants with less drastic consequences on citrin protein formation, such as c.1177+1G>A, are also expected to eliminate citrin activity. Another illustration of this is the c.1018+1G>A variant, currently unregistered in ClinVar, detected in a homozygous state in a AACD patient with impaired consciousness and liver cirrhosis (Data S1).

Citrin consists of a calcium-binding domain (EF1–EF8), carrier domain (H1–H6), and C-terminal domain, as previously reviewed [97]. Even mutations resulting in relatively small C-terminal truncations, such as c.1813C>T and c.1801G>T, could disrupt transmembrane Helix 6, leading to loss of transport function [97]. Therefore, pathogenic variants resulting in the deletion of both carrier domain and C-terminal domain, such as c.852_855del, are expected to have deleterious effects, completely abolishing citrin function. However, the molecular and cellular mechanisms underlying the disease are not always understood, and the metabolic consequences have not been fully elucidated for any of the variants [14, 98, 99]. Exon skipping resulting from variants c.1177+1G>A (IVS11+1G>A), c.1311+1G>A (IVS13+1G>A), and c.1453-1G>A (c.1452+1G>A) does not create a premature stop codon, but could generate a frame-shifted protein, which would also be inactive.

**Figure 4:**
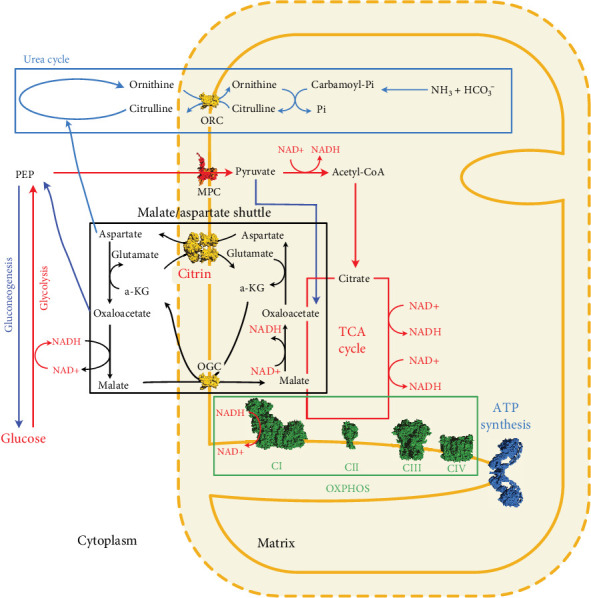
Major metabolic pathways related to citrin function. The malate–aspartate shuttle steps are shown in black, glycolysis and the TCA cycle are featured in red, gluconeogenesis in royal blue, the respiratory chain in green, and the ammonia fixation/urea cycle in light blue. Citrin is shown in yellow together with the oxoglutarate carrier (OGC) and the ornithine carrier (ORC). The mitochondrial pyruvate carrier (MPC) is shown in red. The respiratory chain Complexes 1–4 (CI–CIV) are shown in green and the dimer of ATP synthase in blue.

The aforementioned alterations to the citrin protein not only impact on the function of this protein as part of the malate–aspartate shuttle, but indirectly on the function of many other metabolic pathways, mainly involving energy production (glycolysis, gluconeogenesis, tricarboxylic acid cycle, and beta-oxidation) and nitrogen detoxification (ureagenesis) (Figure 4). Since during the neonatal and infancy period energy requirements per body weight are higher than in later childhood, patients with a defective citrin protein may develop NICCD. As energy requirements decrease towards the end of infancy, patients might seemingly tolerate a citrin defect. However, due to the chronic subclinical progression of liver energy impairment, fatty liver disease may eventually manifest as AACD and progress to hepatic fibrosis, cirrhosis, and, in rare cases, liver cancer. It is reasonable to hypothesize that the progression of the disease as well as the extent of symptoms are at least partly related to the activity levels of the citrin protein in liver mitochondria, suggesting a correlation of genotype and phenotype in this disease.

Another particular aspect of CD concerns the previously reported almost equal male-to-female ratio in NICCD (73:80), but a distorted male-to-female ratio of 2.4:1 (120:50) in AACD [100]. This finding could be confirmed in the present study, in which we likewise found an equal gender ratio in NICCD, but more male than female AACD patients (28:13). The cause of this unexpected distortion in an autosomal recessive condition is thought to result from a difference in eating habits with males consuming more carbohydrate-rich food such as boiled rice and hence demonstrating an increased likelihood to develop AACD when compared to females.

Regarding the most frequently found genetic alterations, the c.852_855del change is generally the most common *SLC25A13* variant in East Asia, while the c.1177+1G>A variant is mainly found in Japan and Korea [5]. For both these changes, the migration pattern explaining their geographic distribution has been described before [97]. It is important to realize that in Japan and most other East Asian countries, rice and products thereof are a major constituent of the daily diet throughout the populations. Such carbohydrate-rich nutrition in individuals with a loss of citrin function, as it must be expected in the background of the aforementioned most prevalent *SLC25A13* variants, will result with great likelihood in the development of any of the phenotypes of CD. Supporting this assumption, most of the CD patients (290 NICCD and 39 AACD) included in this study were in fact reported from Asian countries, and in particular from East Asia. Therefore, a significant impact of these most prevalent variants on patients from East Asian countries can be expected.

Regarding study limitations, some degree of data inhomogeneity was observed when we combined the results from the nationwide study in Japan with the literature review. This was especially due to a shortage of information regarding the clinical manifestations from the literature review, resulting in a predominance of Japanese patients in this study. Moreover, while this study was necessarily retrospective, it would be desirable to collect clinical data in a global, prospective, and systematic way hereby completing and improving data sets.

**Figure 5:**
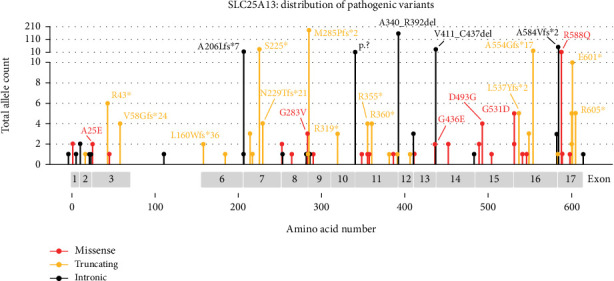
The distribution of pathogenic variants in *SLC25A13*. Lollipop graph showing the distribution of all known SLC25A13 sequence variants depicted with a one-letter code. Missense variants are shown in red, truncating variants in yellow, and intronic variants in black. Exons 4 and 5 of *SLC25A13* carry no reported sequence variants and are therefore not shown on the amino acid bar at the bottom of the figure. The frequency of sequence variants is shown by the length on the lollipop on a partly logarithmic scale.

In conclusion, we report on the mutational background in patients with CD mainly from Japan, but also many other countries (Figure 5). We found certain variants to be highly prevalent in all ethnicities and in all stages of the disease, such as most prominently in the case of c.852_855del. It is suggested that certain pathogenic variants such as c.852_855del often contribute to a more severe long-term clinical outcome. In-depth understanding of the molecular basis of CD could provide useful insight in patient management and pave the way for novel therapeutic strategies, such as gene editing and other gene therapy approaches.

## Figures and Tables

**Table 1:** Variants in *SLC25A13* in 285 patients with NICCD and 19 patients with post-NICCD.

**Variants**	**Allele frequency (%)**
c.852_855del (p.Met285Profs∗2)	157 (27.9)
c.1177+1G>A (p.Val340_Arg392del)	143 (25.4)
IVS16ins3kb^a^	47 (8.4)
c.1311+1G>A (p.Val411_Cys437del)	26 (4.6)
c.674C>A (p.Ser225∗)	26 (4.6)
c.1638_1660dup (p.Ala554Glyfs∗17)	23 (4.1)
c.615+5G>A (p.Ala206Valfs∗7)	14 (2.5)
c.1763G>A (p.Arg588Gln)	11 (2.0)
c.1018+1G>A (p.?)	10 (1.8)
c.1801G>T (p.Glu601∗)	8 (1.4)
c.127C>T (p.Arg43∗)	5 (0.9)
c.1799dup (p.Tyr600∗)	5 (0.9)
c.173_174del (p.Val58Glyfs∗24)	4 (0.7)
c.686del (p.Asn229Thrfs∗21)	4 (0.7)
c.1078C>T (p.Arg360∗)	4 (0.7)
c.640C>T (p.Gln214∗)	3 (0.4)
c.848G>T (p.Gly283Val)	3 (0.5)
c.955C>T (p.Arg319∗)	3 (0.5)
c.1063C>T (p.Arg355∗)	3 (0.5)
c.1230+1G>A (p.?)	3 (0.5)
c.1592G>A (p.Gly531Asp)	3 (0.5)
c.1610_1612delinsAT (p.Leu537Tyrfs∗2)	3 (0.5)
c.1665_1842-32del516 (p.?)	3 (0.5)
c.1813C>T (p.Arg605∗)	3 (0.5)
c.2T>C (p.Met1_Phe34del)	2 (0.4)
c.(69+1_70-1)_(212+1_231-1)del (p.?)	2 (0.4)
c.74C>A (p.Ala25Glu)	2 (0.4)
c.478del (p.Leu160Trpfs∗36)	2 (0.4)
c.754G>A (p.Glu252Lys)	2 (0.4)
c.848+3A>C (p.?)	2 (0.4)
c.848+6T>C (p.?)	2 (0.4)
c.1307_1308delinsAA (p.Gly436Glu)	2 (0.4)
c.1354G>A (p.Val452Ile)	2 (0.4)
c.1465T>C (p.Cys489Arg)	2 (0.4)
c.1800C>G (p.Tyr600∗)	2 (0.4)
c.-3251_15+18443del21709 (p.?)	1 (0.2)
c.15G>A (p.?)	1 (0.2)
c.46G>T (p.Glu16∗)	1 (0.2)
c.69+5G>A (p.Val6_Lys23del)	1 (0.2)
c.70-862_212+3527del4532 (p.Tyr24Ilefs∗11)	1 (0.2)
c.135G>C (p.Leu45Phe)	1 (0.2)
c.329-1687_468+3865del5692 (p.Glu110Glyfs∗18)	1 (0.2)
c.550C>T (p.Arg184∗)	1 (0.2)
c.615+1G>C (p.Ala206Leufs∗7)	1 (0.2)
c.755-2A>G (p.?)	1 (0.2)
c.790G>A (p.Val264Ile)	1 (0.2)
c.847G>T (p.Gly283∗)	1 (0.2)
c.1043C>T (p.Pro348Leu)	1 (0.2)
c.1064G>A (p.Arg355Gln)	1 (0.2)
c.1141del (p.Val381Cysfs∗27)	1 (0.2)
c.1157G>T (p.Gly386Val)	1 (0.2)
c.1173T>G (p.Tyr391∗)	1 (0.2)
c.1216dup (p.Ala406Glyfs∗13)	1 (0.2)
c.1453-1G>A (p.?)	1 (0.2)
c.1511A>G (p.Tyr504Cys)	1 (0.2)
c.1622C>A (p.Ala541Asp)	1 (0.2)
c.1637C>G (p.Thr546Arg)	1 (0.2)
c.1709_1710insA (p.Arg571Alafs2∗)	1 (0.2)
c.1766C>T (p.Ser589Phe)	1 (0.2)
c.1793T>G (p.Leu598Arg)	1 (0.2)
c.1841+3_1841+4del (p.?)	1 (0.2)
**Total**	**562**

*Note:* In 46 patients, only a single variant was detected.

^a^IVS16ins3kb: c.1750_1751 [insNM_138459.3: 2672_24; 1750+72_1751-4dup].

**Table 2:** Variants in *SLC25A13* in 41 patients with AACD.

**Variants**	**Allele frequency (%)**
c.852_855del (p.Met285Profs∗2)	25 (30.5)
c.1177+1G>A (p.Val340_Arg392del)	15 (18.3)
c.674C>A (p.Ser225∗)	7 (8.5)
c.1311+1G>A (p.Val411_Cys437del)	6 (7.3)
c.1478A>G (p.Asp493Gly)	4 (4.9)
IVS16ins3kb^a^	4 (4.9)
c.1645C>T (p.Glu549∗)	3 (3.7)
c.1018+1G>A (p.?)	2 (2.4)
c.1591G>A (p.Gly531Ser)	2 (2.4)
c.1592G>A (p.Gly531Asp)	2 (2.4)
c.1610_1612delinsAT (p.Leu537Tyrfs∗2)	2 (2.4)
c.1801G>T (p.Glu601∗)	2 (2.4)
c.1813C>T (p.Arg605∗)	2 (2.4)
c.127C>T (p.Arg43∗)	1 (1.2)
c.650del (p.Phe217Serfs∗33)	1 (1.2)
c.869T>C (p.Ile290Thr)	1 (1.2)
c.1063C>T (p.Arg355∗)	1 (1.2)
c.1070A>G (p.Gln357Arg)	1 (1.2)
c.1231G>A (p.Val411Met)	1 (1.2)
**Total**	**82**

^a^IVS16ins3kb: c.1750_1751 [insNM_138459.3:2672_24; 1750+72_1751-4dup].

## Data Availability

The data that support the findings of this study are available on request from the corresponding author. The data are not publicly available due to privacy or ethical restrictions.

## General Statement

*Take-home message*. Citrin deficiency is a metabolic liver condition with wide genetic heterogeneity (> 120 pathogenic sequence variants known); few mutations, such as c.852_855del and c.1177+1G>A, are highly prevalent in all age groups and are associated with a significant impact on the clinical outcome in patients.

## Supporting Information

Supporting Information Additional supporting information can be found online in the Supporting Information section. Data S1. Variants in the *SLC25A13* gene in Japan and all literatures (Reference sequence: NG_012247.2, NM_014251.3). Data S2. High-frequent clinical manifestations and variants distribution in the 285 patients with NICCD and 19 patients with post-NICCD. Data S3. Low-frequent clinical manifestations and variants distribution in the 285 patients with NICCD and 19 patients with post-NICCD. Data S4. The relation of nonpresentation of symptoms and variants in the 285 patients with NICCD. Data S5. Clinical manifestations and variants distribution in the 41 patients with AACD.
