# Supplementary material for: Deciphering the Mutational Background in Citrin Deficiency Through a Nationwide Study in Japan and Literature Review
Source: Hum Mutat. 2025 Apr 22;2025:9326326. doi: 10.1155/humu/9326326 (PMC12041640; doi:10.1155/humu/9326326)
Supplement: Supporting Information — Additional supporting information can be found online in the Supporting Information section. Data S1. Variants in the SLC25A13 gene in Japan and all literatures (Reference sequence: NG_012247.2, NM_014251.3). Data S2. High-frequent clinical manifestations and variants distribution in the 285 patients with NICCD and 19 patients with post-NICCD. Data S3. Low-frequent clinical manifestations and variants distribution in the 285 patients with NICCD and 19 patients with post-NICCD. Data S4. The relation of nonpresentation of symptoms and variants in the 285 patients with NICCD. Data S5. Clinical manifestations and variants distribution in the 41 patients with AACD. [file 9326326.f1.docx]

Supplemental data 1. Variants in the *SLC25A13* gene in Japan and all literatures (Reference sequence: NG_012247.2, NM_014251.3)

| Variant No. | Nucleic acid | Amino acid | Location | ClinVar | Polyphen-2 (Score) | SIFT | Frequency (%) | | | Reference |
| --- | --- | --- | --- | --- | --- | --- | --- | --- | --- | --- |
|  |  |  |  |  |  |  | Total | NICCD | CTLN2 |  |
| 1 | c.-3251_16-6548del21709 | p.? |  | NR | - | - | 0.14 (1/690) | 0.16 (1/608) |  | Zhang ZH (2017) |
| 2 | c.2T>C | p.Met1_Phe34del | Exon 1 | Pathogenic, uncertain significance | Possibly damaging (0.462) | Damaging | 0.29 (2/690) | 0.33 (2/608) |  | Zhang (2012),  Zeng (2014) |
| 3 | c.15G>A | p.? | Exon 1 | Pathogenic | - | Damaging | 0.14 (1/690) | 0.16 (1/608) |  |  |
| 4 | c.46G>T | p.Glu16* | Exon 2 | NR | - | Damaging | 0.14 (1/690) | 0.16 (1/608) |  |  |
| 5 | c.(69+1_70-1)_(212+1_231-1)del | p.? |  | NR | - | - | 0.29 (2/690) | 0.33 (2/608) |  | Grunert (2020) |
| 6 | c.69+5G>A | p.Val6_Lys23del | Intron 2 | Pathogenic | - | - | 0.14 (1/690) | 0.16 (1/608) |  | Lin WX (2021) |
| 7 | c.70-862_c.212+3527del4532 | p.Tyr24Ilefs*11 |  | NR | - | - | 0.14 (1/690) | 0.16 (1/608) |  | Wong (2008) |
| 8 | c.74C>A | p.Ala25Glu | Exon 3 | NR | Probably damaging (1.000) | Damaging | 0.29 (2/690) | 0.33 (2/608) |  | Dimmock (2009) |
| 9 | c.127C>T | p.Arg43* | Exon 3 | Pathogenic/likely pathogenic | - | Damaging | 0.87 (6/690) | 0.82 (5/608) | 1.22 (1/82) | Dimmock (2009) |
| 10 | c.135G>C | p.Leu45Phe | Exon 3 | Pathogenic, uncertain significance | Probably damaging (0.997) | Damaging (0.01) | 0.14 (1/690) | 0.16 (1/608) |  | Sun (2023) |
| 11 | c.173_174del | p.Val58Glyfs*24 | Exon 3 | Pathogenic | - | Damaging (0.579) | 0.58 (4/690) | 0.66 (4/608) |  | Dimmock (2009) |
| 12 | c.329-1687_468+3865del5692 | p.Glu110Glyfs*18 |  | NR | - | - | 0.14 (1/690) | 0.16 (1/608) |  | Zheng (2016) |
| 13 | c.478del | p.Leu160Trpfs*36 | Exon 6 | NR | - |  | 0.29 (2/690) | 0.33 (2/608) |  | Kose (2020) |
| 14 | c.550C>T | p.Arg184* | Exon 6 | Pathogenic | - | Damaging | 0.14 (1/690) | 0.16 (1/608) |  | Pinto (2020) |
| 15 | c.615+1G>C | p.Ala206Leufs*7 | Intron 6 | not provided | - | - | 0.14 (1/690) | 0.16 (1/608) |  |  |
| 16 | c.615+5G>A | p.Ala206Valfs*7 | Intron 6 | Pathogenic/likely pathogenic | - | - | 2.03 (14/690) | 2.30 (14/608) |  | Saheki (2004),  Song (2008) |
| 17 | c.640C>T | p.Gln214* | Exon 7 | Pathogenic | - | Damaging | 0.43 (3/690) | 0.49 (3/608) |  | Wang (2015),  Kose (2020) |
| 18 | c.650del | p.Phe217Serfs*33 | Exon 7 | NR | - | Damaging  (0.858) | 0.14 (1/690) |  | 1.22 (1/82) | Bijarnia-Mahay (2015) |
| 19 | c.674C>A | p.Ser225* | Exon 7 | Pathogenic | - | Damaging | 4.78 (33/690) | 4.28 (26/608) | 8.54 (7/82) |  |
| 20 | c.686del | p.Asn229Thrfs*21 | Exon 7 | NR |  | Damaging  (0.858) | 0.58 (4/690) | 0.66 (4/608) |  | Tomé (2021) |
| 21 | c.754G>A | p.Glu252Lys | Exon 7 | Pathogenic/likely pathogenic | Probably damaging (0.981) | Damaging | 0.29 (2/690) | 0.33 (2/608) |  | Lin WX (2012) |
| 22 | c.755-2A>G | p.? | Intron 7 | NR | - | - | 0.14 (1/690) | 0.16 (1/608) |  | Song (2008) |
| 23 | c.790G>A | p.Val264Ile | Exon 8 | Uncertain significance, likely benign | Benign (0.000) | Damaging | 0.14 (1/690) | 0.16 (1/608) |  | Zeng (2014) |
| 24 | c.847G>T | p.Gly283* | Exon 8 | NR | - | Damaging | 0.14 (1/690) | 0.16 (1/608) |  |  |
| 25 | c.848G>T | p.Gly283Val | Exon 8 | NR | Probably damaging (0.996) | Damaging | 0.43 (3/690) | 0.49 (3/608) |  | Pinto (2020) |
| 26 | c.848+3A>C | p.? | Intron 8 | Pathogenic | - | - | 0.29 (2/690) | 0.33 (2/608) |  | Dimmock (2009) |
| 27 | c.848+6T>C | p.? | Intron 8 | NR | - | - | 0.29 (2/690) | 0.33 (2/608) |  | Sachs (2023) |
| 28 | c.852_855del | p.Mst285Profs*2 | Exon 9 | Pathogenic | - | Damaging (0.529) | 26.38 (182/690) | 25.82 (157/608) | 30.49 (25/82) |  |
| 29 | c.869T>C | p.Ile290Thr | Exon 9 | NR | Probably damaging (0.965) | Damaging | 0.14 (1/690) |  | 1.22 (1/82) | Bijarnia-Mahay (2015) |
| 30 | c.955C>T | p.Arg319* | Exon 10 | Pathogenic | - | Damaging | 0.43 (3/690) | 0.49 (3/608) |  |  |
| 31 | c.1018+1G>A | p.? | Intron 10 | NR | - | - | 1.74 (12/690) | 1.64 (10/608) | 2.44 (2/82) |  |
| 32 | c.1043C>T | p.Pro348Leu | Exon 11 | NR | Probably damaging (1.000) | Damaging | 0.14 (1/690) | 0.16 (1/608) |  | Sun (2023) |
| 33 | c.1063C>T | p.Arg355* | Exon 11 | Pathogenic, uncertain significance | - | Damaging | 0.58 (4/690) | 0.49 (3/608) | 1.22 (1/82) | Dimmock (2009) |
| 34 | c.1064G>A | p.Arg355Gln | Exon 11 | Pathogenic, likely pathogenic, uncertain significance | Probably damaging (1.000) | Damaging | 0.14 (1/690) | 0.16 (1/608) |  | Zhang ZH (2014) |
| 35 | c.1070A>G | p.Gln357Arg | Exon 11 | NR | Probably damaging (1.000) | Damaging | 0.14 (1/690) |  | 1.22 (1/82) | Yazaki (2012) |
| 36 | c.1078C>T | p.Arg360* | Exon 11 | Pathogenic/Likely pathogenic | - | Damaging | 0.58 (4/690) | 0.66 (4/608) |  |  |
| 37 | c.1141del | p.Val381Cysfs*27 | Exon 11 | NR | - | Damaging  (0.858) | 0.14 (1/690) | 0.16 (1/608) |  | Wang (2023) |
| 38 | c.1157G>T | p.Gly386Val | Exon 11 | NR | Probably damaging (1.000) | Damaging | 0.14 (1/690) | 0.16 (1/608) |  | Wang XX (2021) |
| 39 | c.1173T>G | p.Tyr391* | Exon 11 | Pathogenic | - | Damaging | 0.14 (1/690) | 0.16 (1/608) |  | Pinto (2020) |
| 40 | c.1177+1G>A | p.Ala340_Arg392del | Intron 11 | Pathogenic | - | - | 22.90 (158/690) | 23.52 (143/608) | 18.29 (15/82) |  |
| 41 | c.1216dup | p.Ala406Glyfs*13 | Exon 12 | NR | - | Damaging (0.858) | 0.14 (1/690) | 0.16 (1/608) |  | Sun (2023) |
| 42 | c.1230+1G>A | p.? | Intron 12 | NR | - | - | 0.43 (3/690) | 0.49 (3/608) |  |  |
| 43 | c.1231G>A | p.Val411Met | Exon 13 | Pathogenic/likely pathogenic | Benign (0.021) | Damaging | 0.14 (1/690) |  | 1.22 (1/82) | Ng (2011) |
| 44 | c.1307_1308delinsAA | p.Gly436Glu | Exon 13 | Likely pathogenic | Probably damaging (0.999) | Damaging (0.529) | 0.29 (2/690) | 0.33 (2/608) |  | Fiermonte (2011) |
| 45 | c.1311+1G>A | p.Val411_Cys437del | Intron 13 | Pathogenic | - | - | 0.72 (32/690) | 4.28 (26/608) | 7.32 (6/82) | Fukushima (2010) |
| 46 | c.1354G>A | p.Val452Ile | Exon 14 | Uncertain significance, benign | Probably damaging (1.000) | Damaging | 0.29 (2/690) | 0.33 (2/608) |  | Seker-Yilmaz (2017) |
| 47 | c.1453-1G>A | p.? | Intron 14 | Pathogenic | - | - | 0.14 (1/690) | 0.16 (1/608) |  | Lin WX (2021) |
| 48 | c.1465T>C | p.Cys489Arg | Exon 15 | NR | Probably damaging (1.000) | Damaging | 0.29 (2/690) | 0.33 (2/608) |  | Hutchin (2009) |
| 49 | c.1478A>G | p.Asp493Gly | Exon 15 | NR | Probably damaging (1.000) | Damaging | 0.58 (4/690) |  | 4.88 (4/82) |  |
| 50 | c.1511A>G | p.Tyr504Cys | Exon 15 | NR | Probably damaging (1.000) | Damaging | 0.14 (1/690) | 0.16 (1/608) |  |  |
| 51 | c.1591G>A | p.Gly531Ser | Exon 15 | NR | Probably damaging (0.999) | Damaging | 0.29 (2/690) |  | 2.44 (2/82) | Baskar (2023) |
| 52 | c.1592G>A | p.Gly531Asp | Exon 16 | Pathogenic | Probably damaging (1.000) | - | 0.72 (5/690) | 0.49 (3/608) | 2.44 (2/82) |  |
| 53 | c.1610_1612delinsAT | p.Leu537Tyrfs*2 | Exon 16 | NR | - | Damaging (0.858) | 0.72 (5/690) | 0.49 (3/608) | 2.44 (2/82) | Hutchin (2009) |
| 54 | c.1622C>A | p.Ala541Asp | Exon 16 | NR | Probably damaging (1.000) | Damaging | 0.14 (1/690) | 0.16 (1/608) |  | Song (2008) |
| 55 | c.1637C>G | p.Thr546Arg | Exon 16 | Uncertain significance | Probably damaging (1.000) | Damaging | 0.14 (1/690) | 0.16 (1/608) |  | Dimmock (2009) |
| 56 | c.1638_1660dup | p.Ala554Glyfs*17 | Exon 16 | Pathogenic | - | Damaging (0.858) | 3.33 (23/690) | 3.78 (23/608) |  |  |
| 57 | c.1645C>T | p.Gln549* | Exon 16 | NR |  | Damaging | 0.43 (3/690) |  | 3.66 (3/82) |  |
| 58 | c.1665_1842-32del516 | p.? | Exon 16 – Intron 16 | NR | - | - | 0.43 (3/690) | 0.49 (3/608) |  | Takaya (2005) |
| 59 | c.1709_1710insA | p.Arg571Alafs2* | Exon 16 | NR | - |  | 0.14 (1/690) | 0.16 (1/608) |  | Wang (2015) |
| 60 | IVS16ins3kb | p.Ala584Valfs*2 | Intron 16 | Pathogenic | - | - | 7.39 (51/690) | 7.73 (47/608) | 4.88 (4/82) |  |
| 61 | c.1763G>A | p.Arg588Gln | Exon 17 | Pathogenic, likely pathogenic, uncertain significance | Probably damaging (0.999) | Damaging | 1.59 (11/690) | 1.81 (11/608) |  | Tabata (2008),  Fiermonte (2008),  Pinto (2020) |
| 62 | c.1766C>T | p.Ser589Phe | Exon 17 | NR | Possibly damaging (0.931) | Damaging | 0.14 (1/690) | 0.16 (1/608) |  | Pinto (2020) |
| 63 | c.1793T>G | p.Leu598Arg | Exon 17 | NR | Possibly damaging (0.573) | Damaging | 0.14 (1/690) | 0.16 (1/608) |  |  |
| 64 | c.1799dup | p.Tyr600* | Exon 17 | Pathogenic | - | Damaging (0.858) | 0.72 (5/690) | 0.82 (5/608) |  |  |
| 65 | c.1800C>G | p.Tyr600* | Exon 17 | NR | - | Damaging | 0.29 (2/690) | 0.33 (2/608) |  | Wang (2023) |
| 66 | c.1801G>T | p.Glu601* | Exon 17 | Pathogenic | - | Damaging | 1.45 (10/690) | 1.32 (8/608) | 2.44 (2/82) |  |
| 67 | c.1813C>T | p.Arg605* | Exon 17 | Pathogenic/Likely pathogenic | - | Damaging | 0.72 (5/690) | 0.49 (3/608) | 2.44 (2/82) |  |
| 68 | c.1841+3_1841+4del | p.? | Intron 17 | Likely pathogenic | - | - | 0.14 (1/690) | 0.16 (1/608) |  | Zhang L (2019) |

NR: not registered, IVS16ins3kb: c.1750_1751[insNM_138459.3:2672_24;1750+72_1751-4dup] (Tabata 2008), IVS4ins6kb: Genbank accession No. KF425758 (Song 2013)

Supplemental data 2. .High-frequent clinical manifestations and variants distribution in the 285 patients with NICCD and 19 patients with post-NICCD

A. Cholestasis (N=132)

| Allele 1 | Allele 2 | # Patients | Study |
| --- | --- | --- | --- |
| c.46G>T (p.Glu16*) | c.852_855del (p.Met285Profs*2) | 1 | Our study |
| c.615+1G>C (p.Ala206Leufs*7) | ND | 1 | Our study |
| c.674C>A (p.Ser225*) | c.852_855del (p.Met285Profs*2) | 3 | Our study |
| c.674C>A (p.Ser225*) | c.1177+1G>A (p.Val340_Arg392del) | 7 | Our study |
| c.674C>A (p.Ser225*) | c.1638_1660dup (p.Ala554Glyfs*17) | 1 | Our study |
| c.674C>A (p.Ser225*) | IVS16ins3kb | 1 | Our study |
| c.674C>A (p.Ser225*) | c.1799dup (p.Tyr600*) | 3 | Our study |
| c.674C>A (p.Ser225*) | ND | 1 | Our study |
| c.852_855del (p.Met285Profs*2) | c.852_855del (p.Met285Profs*2) | 10 | Our study |
| c.852_855del (p.Met285Profs*2) | c.1018+1G>A (p.?) | 1 | Our study |
| c.852_855del (p.Met285Profs*2) | c.1177+1G>A (p.Val340_Arg392del) | 26 | Our study |
| c.852_855del (p.Met285Profs*2) | c.1078C>T (p.Arg360*) | 1 | Our study |
| c.852_855del (p.Met285Profs*2) | c.1230+1G>A (p.?) | 1 | Our study |
| c.852_855del (p.Met285Profs*2) | c.1311+1G>A (p.Val411_Cys437del) | 4 | Our study |
| c.852_855del (p.Met285Profs*2) | c.1638_1660dup (p.Ala554Glyfs*17) | 1 | Our study |
| c.852_855del (p.Met285Profs*2) | IVS16ins3kb | 2 | Our study |
| c.852_855del (p.Met285Profs*2) | c.1799dupA (p.Tyr600*) | 2 | Our study |
| c.852_855del (p.Met285Profs*2) | c.1801G>T (p.Glu601*) | 1 | Our study |
| c.852_855del (p.Met285Profs*2) | ND | 5 | Our study |
| c.1018+1G>A (p.?) | c.1018+1G>A (p.?) | 2 | Our study |
| c.1018+1G>A (p.?) | c.1638_1660dup (p.Ala554Glyfs*17) | 1 | Our study |
| c.1177+1G>A (p.Val340_Arg392del) | c.1177+1G>A (p.Val340_Arg392del) | 24 | Our study |
| c.1177+1G>A (p.Val340_Arg392del) | c.1311+1G>A (p.Val411_Cys437del) | 7 | Our study |
| c.1177+1G>A (p.Val340_Arg392del) | c.1511A>G (p.Tyr504Cys) | 1 | Our study |
| c.1177+1G>A (p.Val340_Arg392del) | c.1592G>A (p.Gly531Asp) | 2 | Our study |
| c.1177+1G>A (p.Val340_Arg392del) | c.1638_1660dup (p.Ala554Glyfs*17) | 3 | Our study |
| c.1177+1G>A (p.Val340_Arg392del) | IVS16ins3kb | 5 | Our study |
| c.1177+1G>A (p.Val340_Arg392del) | c.1801G>T (p.Glu601*) | 1 | Our study |
| c.1177+1G>A (p.Val340_Arg392del) | ND | 4 | Our study |
| c.1177+1G>A (p.Val340_Arg392del) | c.1813C>T (p.Arg605*) | 1 | Our study |
| c.1230+1G>A (p.?) | IVS16ins3kb | 1 | Our study |
| c.1311+1G>A (p.Val411_Cys437del) | c.1311+1G>A (p.Val411_Cys437del) | 3 | Our study |
| c.1311+1G>A (p.Val411_Cys437del) | IVS16ins3kb | 2 | Our study |
| c.1311+1G>A (p.Val411_Cys437del) | c.1801G>T (p.Glu601*) | 1 | Our study |
| c.1311+1G>A (p.Val411_Cys437del) | ND | 1 | Our study |
| c.1638_1660dup (p.A554Gfs*17) | ND | 1 | Our study |

IVS16ins3kb: c.1750_1751[insNM_138459.3:2672_24;1750+72_1751-4dup], ND: not detectable

B. Elevated transaminase (≥100 U/L) (N=74)

| Allele 1 | Allele 2 | # Patients | Study |
| --- | --- | --- | --- |
| c.15G>A (p.K5=) | c.1177+1G>A (p.V340_R392del) | 1 | Our study |
| c.46G>T (p.Glu16*) | c.852_855del (p.Met285Profs*2) | 1 | Our study |
| c.674C>A (p.Ser225*) | c.852_855del (p.Met285Profs*2) | 2 | Our study |
| c.674C>A (p.Ser225*) | c.1177+1G>A (p.Val340_Arg392del) | 3 | Our study |
| c.674C>A (p.Ser225*) | IVS16ins3kb | 1 | Our study |
| c.674C>A (p.Ser225*) | c.1799dupA (p.Tyr600*) | 1 | Our study |
| c.852_855del (p.Met285Profs*2) | c.852_855del (p.Met285Profs*2) | 8 | Our study |
| c.852_855del (p.Met285Profs*2) | c.1078C>T (p.Arg360*) | 1 | Our study |
| c.852_855del (p.Met285Profs*2) | c.1177+1G>A (p.Val340_Arg392del) | 16 | Our study |
| c.852_855del (p.Met285Profs*2) | c.1230+1G>A (p.?) | 1 | Our study |
| c.852_855del (p.Met285Profs*2) | c.1311+1G>A (p.Val411_Cys437del) | 2 | Our study |
| c.852_855del (p.Met285Profs*2) | c.1638_1660dup (p.Ala554Glyfs*17) | 2 | Our study |
| c.852_855del (p.Met285Profs*2) | IVS16ins3kb | 1 | Our study |
| c.852_855del (p.Met285Profs*2) | c.1799dupA (p.Tyr600*) | 1 | Our study |
| c.852_855del (p.Met285Profs*2) | c.1801G>T (p.Glu601*) | 1 | Our study |
| c.852_855del (p.Met285Profs*2) | ND | 2 | Our study |
| c.1018+1G>A (p.?) | c.1018+1G>A (p.?) | 1 | Our study |
| c.1177+1G>A (p.Val340_Arg392del) | c.1177+1G>A (p.Val340_Arg392del) | 8 | Our study |
| c.1177+1G>A (p.Val340_Arg392del) | c.1311+1G>A (p.Val411_Cys437del) | 4 | Our study |
| c.1177+1G>A (p.Val340_Arg392del) | c.1592G>A (p.Gly531Asp) | 2 | Our study |
| c.1177+1G>A (p.Val340_Arg392del) | c.1638_1660dup (p.Ala554Glyfs*17) | 2 | Our study |
| c.1177+1G>A (p.Val340_Arg392del) | IVS16ins3kb | 3 | Our study |
| c.1177+1G>A (p.Val340_Arg392del) | c.1801G>T (p.Glu601*) | 2 | Our study |
| c.1177+1G>A (p.Val340_Arg392del) | ND | 1 | Our study |
| c.1230+1G>A (p.?) | IVS16ins3kb | 1 | Our study |
| c.1311+1G>A (p.Val411_Cys437del) | c.1311+1G>A (p.Val411_Cys437del) | 3 | Our study |
| c.1311+1G>A (p.Val411_Cys437del) | IVS16ins3kb | 1 | Our study |
| c.1311+1G>A (p.Val411_Cys437del) | c.1801G>T (p.Glu601*) | 1 | Our study |
| c.1311+1G>A (p.Val411_Cys437del) | ND | 1 | Our study |

IVS16ins3kb: c.1750_1751[insNM_138459.3:2672_24;1750+72_1751-4dup], ND: not detectable

C. Fatty liver (N=58)

| Allele 1 | Allele 2 | # Patients | Study |
| --- | --- | --- | --- |
| c.615+5G>A (p.Ala206Valfs*7) | c.852_855del (p.Met285Profs*2) | 1 | Our study |
| c.674C>A (p.Ser225*) | c.852_855del (p.Met285Profs*2) | 1 | Our study |
| c.674C>A (p.Ser225*) | c.1177+1G>A (p.Val340_Arg392del) | 2 | Our study |
| c.674C>A (p.Ser225*) | c.1799dupA (p.Tyr600*) | 2 | Our study |
| c.852_855del (p.Met285Profs*2) | c.852_855del (p.Met285Profs*2) | 3 | Our study |
| c.852_855del (p.Met285Profs*2) | c.1078C>T (p.Arg360*) | 1 | Our study |
| c.852_855del (p.Met285Profs*2) | c.1177+1G>A (p.Val340_Arg392del) | 9 | Our study |
| c.852_855del (p.Met285Profs*2) | c.1230+1G>A (p.?) | 1 | Our study |
| c.852_855del (p.Met285Profs*2) | c.1311+1G>A (p.Val411_Cys437del) | 2 | Our study |
| c.852_855del (p.Met285Profs*2) | IVS16ins3kb | 2 | Our study |
| c.852_855del (p.Met285Profs*2) | c.1801G>T (p.Glu601*) | 1 | Our study |
| c.852_855del (p.Met285Profs*2) | ND | 2 | Our study |
| c.955C>T (p.Arg319*) | c.1177+1G>A (p.Val340_Arg392del) | 1 | Our study |
| c.1177+1G>A (p.Val340_Arg392del) | c.1177+1G>A (p.Val340_Arg392del) | 8 | Our study |
| c.1177+1G>A (p.Val340_Arg392del) | c.1311+1G>A (p.Val411_Cys437del) | 7 | Our study |
| c.1177+1G>A (p.Val340_Arg392del) | c.1511A>G (p.Tyr504Cys) | 1 | Our study |
| c.1177+1G>A (p.Val340_Arg392del) | c.1638_1660dup (p.Ala554Glyfs*17) | 3 | Our study |
| c.1177+1G>A (p.Val340_Arg392del) | c.1801G>T (p.Glu601*) | 1 | Our study |
| c.1177+1G>A (p.Val340_Arg392del) | ND | 2 | Our study |
| c.1177+1G>A (p.Val340_Arg392del) | c.1813C>T (p.Arg605*) | 1 | Our study |
| c.1311+1G>A (p.Val411_Cys437del) | c.1311+1G>A (p.Val411_Cys437del) | 1 | Our study |
| c.1311+1G>A (p.Val411_Cys437del) | IVS16ins3kb | 2 | Our study |
| c.1311+1G>A (p.Val411_Cys437del) | c.1801G>T (p.Glu601*) | 1 | Our study |
| c.1453-1G>A (p.?) | IVS16ins3kb | 1 | Lin WX (2021) |
| c.1638_1660dup (p.Ala554Glyfs*17) | c.1800C>G (p.Tyr600*) | 1 | Wang (2023) |
| IVS16ins3kb | c.1800C>G (p.Tyr600*) | 1 | Wang (2023) |

IVS16ins3kb: c.1750_1751[insNM_138459.3:2672_24;1750+72_1751-4dup], ND: not detectable

D. Hyperlipidemia (N=41)

| Allele 1 | Allele 2 | # Patients | Study |
| --- | --- | --- | --- |
| c.615+1G>C (p.Ala206Leufs*7) | ND | 1 | Our study |
| c.674C>A (p.Ser225*) | c.852_855del (p.Met285Profs*2) | 2 | Our study |
| c.674C>A (p.Ser225*) | c.1177+1G>A (p.Val340_Arg392del) | 2 | Our study |
| c.674C>A (p.Ser225*) | IVS16ins3kb | 1 | Our study |
| c.674C>A (p.Ser225*) | c.1799dupA (p.Tyr600*) | 1 | Our study |
| c.852_855del (p.Met285Profs*2) | c.852_855del (p.Met285Profs*2) | 4 | Our study |
| c.852_855del (p.Met285Profs*2) | c.1177+1G>A (p.Val340_Arg392del) | 6 | Our study |
| c.852_855del (p.Met285Profs*2) | c.1311+1G>A (p.Val411_Cys437del) | 2 | Our study |
| c.852_855del (p.Met285Profs*2) | c.1638_1660dup (p.Ala554Glyfs*17) | 1 | Our study |
| c.852_855del (p.Met285Profs*2) | c.1799dupA (p.Tyr600*) | 1 | Our study |
| c.852_855del (p.Met285Profs*2) | c.1801G>T (p.Glu601*) | 1 | Our study |
| c.1018+1G>A (p.?) | c.1018+1G>A (p.?) | 1 | Our study |
| c.1177+1G>A (p.Val340_Arg392del) | c.1177+1G>A (p.Val340_Arg392del) | 3 | Our study |
| c.1177+1G>A (p.Val340_Arg392del) | c.1311+1G>A (p.Val411_Cys437del) | 2 | Our study |
| c.1177+1G>A (p.Val340_Arg392del) | c.1511A>G (p.Tyr504Cys) | 1 | Our study |
| c.1177+1G>A (p.Val340_Arg392del) | c.1592G>A (p.Gly531Asp) | 1 | Our study |
| c.1177+1G>A (p.Val340_Arg392del) | c.1638_1660dup (p.Ala554Glyfs*17) | 1 | Our study |
| c.1177+1G>A (p.Val340_Arg392del) | IVS16ins3kb | 2 | Our study |
| c.1177+1G>A (p.Val340_Arg392del) | c.1813C>T (p.Arg605*) | 1 | Our study,  Wada (2020) |
| c.1177+1G>A (p.Val340_Arg392del) | ND | 1 | Our study |
| c.1230+1G>A (p.?) | IVS16ins3kb | 2 | Our study |
| c.1311+1G>A (p.Val411_Cys437del) | c.1311+1G>A (p.Val411_Cys437del) | 2 | Our study |
| c.1311+1G>A (p.Val411_Cys437del) | IVS16ins3kb | 1 | Our study |
| c.1311+1G>A (p.Val411_Cys437del) | c.1801G>T (p.Glu601*) | 1 | Our study |

IVS16ins3kb: c.1750_1751[insNM_138459.3:2672_24;1750+72_1751-4dup], ND: not detectable

E. Hypoglycemia (N=53)

| Allele 1 | Allele 2 | # Patiens | Study |
| --- | --- | --- | --- |
| c.46G>T (p.Glu16*) | c.852_855del (p.Met285Profs*2) | 1 | Our study |
| c.615+1G>C (p.Ala206Leufs*7) | ND | 1 | Our study |
| c.674C>A (p.Ser225*) | c.852_855del (p.Met285Profs*2) | 3 | Our study |
| c.674C>A (p.Ser225*) | c.1177+1G>A (p.Val340_Arg392del) | 4 | Our study |
| c.674C>A (p.Ser225*) | IVS16ins3kb | 1 | Our study |
| c.674C>A (p.Ser225*) | ND | 2 | Our study |
| c.852_855del (p.Met285Profs*2) | c.852_855del (p.Met285Profs*2) | 4 | Our study |
| c.852_855del (p.Met285Profs*2) | c.1177+1G>A (p.Val340_Arg392del) | 9 | Our study |
| c.852_855del (p.Met285Profs*2) | c.1311+1G>A (p.Val411_Cys437del) | 2 | Our study |
| c.852_855del (p.Met285Profs*2) | c.1638_1660dup (p.Ala554Glyfs*17) | 1 | Our study |
| c.852_855del (p.Met285Profs*2) | c.1793T>G (p.Leu598Arg) | 1 | Our study |
| c.852_855del (p.Met285Profs*2) | c.1801G>T (p.Glu601*) | 1 | Our study |
| c.852_855del (p.Met285Profs*2) | ND | 2 | Our study |
| c.1018+1G>A (p.?) | c.1018+1G>A (p.?) | 1 | Our study |
| c.1177+1G>A (p.Val340_Arg392del) | c.1177+1G>A (p.Val340_Arg392del) | 5 | Our study |
| c.1177+1G>A (p.Val340_Arg392del) | c.1311+1G>A (p.Val411_Cys437del) | 6 | Our study |
| c.1177+1G>A (p.Val340_Arg392del) | c.1511A>G (p.Tyr504Cys) | 1 | Our study |
| c.1177+1G>A (p.Val340_Arg392del) | c.1638_1660dup (p.Ala554Glyfs*17) | 2 | Our study |
| c.1177+1G>A (p.Val340_Arg392del) | IVS16ins3kb | 1 | Our study |
| c.1177+1G>A (p.Val340_Arg392del) | c.1801G>T (p.Glu601*) | 1 | Our study |
| c.1177+1G>A (p.Val340_Arg392del) | c.1813C>T (p.Arg605*) | 1 | Our study, Wada (2020) |
| c.1177+1G>A (p.Val340_Arg392del) | ND | 1 | Our study |
| c.1311+1G>A (p.Val411_Cys437del) | c.1311+1G>A (p.Val411_Cys437del) | 1 | Our study |
| c.1311+1G>A (p.Val411_Cys437del) | c.1801G>T (p.Glu601*) | 1 | Our study |

IVS16ins3kb: c.1750_1751[insNM_138459.3:2672_24;1750+72_1751-4dup], ND: not detectable

F. Hyperammonemia（≥ 180 umol/L）(N=61)

| Allele 1 | Allele 2 | # Patients | Study |
| --- | --- | --- | --- |
| c.46G>T (p.Glu16*) | c.852_855del (p.Met285Profs*2) | 1 | Our study |
| c.478del (p.Leu160Trpfs*36) | c.478del (p.Leu160Trpfs*36) | 1 | Kose (2020) |
| c.615+5G>A (p.Ala206Valfs*7) | c.852_855del (p.Met285Profs*2) | 2 | Wang XX (2021), Dimmock (2009) |
| c.615+5G>A (p.Ala206Valfs*7) | IVS16ins3kb | 1 | Wang XX (2021) |
| c.640C>T (p.Gln214*) | c.640C>T (p.Gln214*) | 1 | Kose (2020) |
| c.640C>T (p.Gln214*) | c.1709_1710insA (p.Arg571Alafs2*) | 1 | Wang (2015) |
| c.674C>A (p.Ser225*) | c.674C>A (p.Ser225*) | 1 | Tamamori (2002) |
| c.674C>A (p.Ser225*) | c.852_855del (p.Met285Profs*2) | 3 | Our study, Ko (2007) |
| c.674C>A (p.Ser225*) | c.1177+1G>A (p.Val340_Arg392del) | 2 | Our study |
| c.674C>A (p.Ser225*) | c.1638_1660dup (p.Ala554Glyfs*17) | 2 | Our study, Ko (2007) |
| c.674C>A (p.Ser225*) | IVS16ins3kb | 1 | Our study |
| c.674C>A (p.Ser225*) | c.1799dupA (p.Tyr600*) | 2 | Our study |
| c.852_855del (p.Met285Profs*2) | c.852_855del (p.Met285Profs*2) | 4 | Our study,  Chew (2010) |
| c.852_855del (p.Met285Profs*2) | c.1177+1G>A (p.Val340_Arg392del) | 9 | Our study  Tamamori (2002), |
| c.852_855del (p.Met285Profs*2) | c.1157C>T (p.Gly386Val) | 1 | Wang XX (2021) |
| c.852_855del (p.Met285Profs*2) | c.1311+1G>A (p.Val411_Cys437del) | 2 | Our study |
| c.852_855del (p.Met285Profs*2) | c.1638_1660dup (p.Ala554Glyfs*17) | 1 | Our study |
| c.852_855del (p.Met285Profs*2) | IVS16ins3kb | 1 | Our study |
| c.852_855del (p.Met285Profs*2) | c.1801G>T (p.Glu601*) | 1 | Our study |
| c.852_855del (p.Met285Profs*2) | c.1177+1G>A (p.Val340_Arg392del) | 1 | Wang (2023) |
| c.852_855del (p.Met285Profs*2) | ND | 2 | Chew (2010),  Tamamori (2002) |
| c.1141delG (p.Val381Cysfs*27) | IVS16ins3kb | 1 | Wang (2023) |
| c.1177+1G>A (p.Val340_Arg392del) | c.1177+1G>A (p.Val340_Arg392del) | 7 | Our study |
| c.1177+1G>A (p.Val340_Arg392del) | c.1311+1G>A (p.Val411_Cys437del) | 3 | Our study |
| c.1177+1G>A (p.Val340_Arg392del) | c.1801G>T (p.Glu601*) | 1 | Tamamori (2002) |
| c.1177+1G>A (p.Val340_Arg392del) | c.1813C>T (p.Arg605*) | 1 | Our study |
| c.1177+1G>A (p.Val340_Arg392del) | ND | 2 | Our study |
| c.1307_1308delinsAA (p.Gly436Glu) | c.1307_1308delinsAA (p.Gly436Glu) | 1 | Fiermonte (2011) |
| c.1311+1G>A (p.Val411_Cys437del) | c.1801G>T (p.Glu601*) | 1 | Our study |
| c.1638_1660dup (p.Ala554Glyfs*17) | ND | 2 | Our study,  Ko (2007) |
| IVS16ins3kb | IVS16ins3kb | 2 | Zhang MH (2015),  Chew (2010) |

IVS16ins3kb: c.1750_1751[insNM_138459.3:2672_24;1750+72_72_1751-4dup], ND: not detectable

G. Hypoproteinemia (N=63)

| Allele 1 | Allele 2 | # Patients | Study |
| --- | --- | --- | --- |
| c.46G>T (p.Glu16*) | c.852_855del (p.Met285Profs*2) | 1 | Our study |
| c.674C>A (p.Ser225*) | c.852_855del (p.Met285Profs*2) | 3 | Our study |
| c.674C>A (p.Ser225*) | c.1177+1G>A (p.Val340_Arg392del) | 5 | Our study |
| c.674C>A (p.Ser225*) | c.1638_1660dup (p.Ala554Glyfs*17) | 1 | Our study |
| c.674C>A (p.Ser225*) | IVS16ins3kb | 1 | Our study |
| c.674C>A (p.Ser225*) | c.1799dupA (p.Tyr600*) | 2 | Our study |
| c.852_855del (p.Met285Profs*2) | c.852_855del (p.Met285Profs*2) | 6 | Our study |
| c.852_855del (p.Met285Profs*2) | c.1177+1G>A (p.Val340_Arg392del) | 10 | Our study |
| c.852_855del (p.Met285Profs*2) | c.1078C>T (p.Arg360*) | 1 | Our study |
| c.852_855del (p.Met285Profs*2) | c.1638_1660dup (p.Ala554Glyfs*17) | 1 | Our study |
| c.852_855del (p.Met285Profs*2) | IVS16ins3kb | 1 | Our study |
| c.852_855del (p.Met285Profs*2) | c.1793T>G (p.Leu598Arg) | 1 | Our study |
| c.852_855del (p.Met285Profs*2) | c.1801G>T (p.Glu601*) | 1 | Wang (2023) |
| c.852_855del (p.Met285Profs*2) | ND | 2 | Our study |
| c.1018+1G>A (p.?) | c.1018+1G>A (p.?) | 2 | Our study |
| c.1141delG (p.Val381Cysfs*27) | IVS16ins3kb | 1 | Wang (2023) |
| c.1177+1G>A (p.Val340_Arg392del) | c.1177+1G>A (p.Val340_Arg392del) | 11 | Our study |
| c.1177+1G>A (p.Val340_Arg392del) | c.1311+1G>A (p.Val411_Cys437del) | 6 | Our study |
| c.1177+1G>A (p.Val340_Arg392del) | c.1511A>G (p.Tyr504Cys) | 1 | Our study |
| c.1177+1G>A (p.Val340_Arg392del) | c.1638_1660dup (p.Ala554Glyfs*17) | 1 | Our study |
| c.1177+1G>A (p.Val340_Arg392del) | c.1801G>T (p.Glu601*) | 1 | Our study |
| c.1177+1G>A (p.Val340_Arg392del) | ND | 1 | Our study |
| c.1230+1G>A (p.?) | IVS16ins3kb | 1 | Our study |
| c.1311+1G>A (p.Val411_Cys437del) | IVS16ins3kb | 1 | Our study |
| c.1311+1G>A (p.Val411_Cys437del) | ND | 1 | Our study |

IVS16ins3kb: c.1750_1751[insNM_138459.3:2672_24;1750+72_1751-4dup], ND: not detectable

H. Anemia (N=29)

| Allele 1 | Allele 2 | # Patients | Study |
| --- | --- | --- | --- |
| c.674C>A (p.Ser225*) | c.852_855del (p.Met285Profs*2) | 2 | Our study |
| c.674C>A (p.Ser225*) | c.1177+1G>A (p.Val340_Arg392del) | 1 | Our study |
| c.674C>A (p.Ser225*) | c.1638_1660dup (p.Ala554Glyfs*17) | 1 | Our study |
| c.674C>A (p.Ser225*) | c.1799dupA (p.Tyr600*) | 2 | Our study |
| c.852_855del (p.Met285Profs*2) | c.1177+1G>A (p.Val340_Arg392del) | 2 | Our study |
| c.852_855del (p.Met285Profs*2) | c.1078C>T (p.Arg360*) | 1 | Our study |
| c.852_855del (p.Met285Profs*2) | c.1311+1G>A (p.Val411_Cys437del) | 2 | Our study |
| c.852_855del (p.Met285Profs*2) | c.1793 T>G (p.Leu598Arg) | 1 | Our study |
| c.852_855del (p.Met285Profs*2) | ND | 2 | Our study |
| c.1018+1G>A (p.?) | c.1638_1660dup (p.Ala554Glyfs*17) | 1 | Our study |
| c.1177+1G>A (p.Val340_Arg392del) | c.1177+1G>A (p.Val340_Arg392del) | 7 | Our study |
| c.1177+1G>A (p.Val340_Arg392del) | c.1311+1G>A (p.Val411_Cys437del) | 2 | Our study |
| c.1177+1G>A (p.Val340_Arg392del) | ND | 2 | Our study |
| c.1230+1G>A (p.?) | IVS16ins3kb | 1 | Our study |
| c.1311+1G>A (p.Val411_Cys437del) | IVS16ins3kb | 2 | Our study |

IVS16ins3kb: c.1750_1751[insNM_138459.3:2672_24;1750+72_1751-4dup], ND: not detectable

I. Prolonged prothrombin time (N=65)

| Allele 1 | Allele 2 | # Patiens | Study |
| --- | --- | --- | --- |
| c.478delC (p.Leu160Trpfs*36) | c.478delC (p.Leu160Trpfs*36) | 1 | Kose (2020) |
| c.674C>A (p.Ser225*) | c.852_855del (p.Met285Profs*2) | 1 | Our study |
| c.674C>A (p.Ser225*) | c.1177+1G>A (p.Val340_Arg392del) | 2 | Our study |
| c.674C>A (p.Ser225*) | c.1638_1660dup (p.Ala554Glyfs*17) | 1 | Our study |
| c.674C>A (p.Ser225*) | IVS16ins3kb | 1 | Our study |
| c.674C>A (p.Ser225*) | c.1799dupA (p.Tyr600*) | 2 | Our study |
| c.852_855del (p.Met285Profs*2) | c.852_855del (p.Met285Profs*2) | 4 | Our study |
| c.852_855del (p.Met285Profs*2) | c.1177+1G>A (p.Val340_Arg392del) | 14 | Our study |
| c.852_855del (p.Met285Profs*2) | c.1311+1G>A (p.Val411_Cys437del) | 1 | Our study |
| c.852_855del (p.Met285Profs*2) | c.1638_1660dup (p.Ala554Glyfs*17) | 1 | Our study |
| c.852_855del (p.Met285Profs*2) | ND | 5 | Our study |
| c.1018+1G>A (p.?) | c.1018+1G>A (p.?) | 1 | Our study |
| c.1078C>T (p.Arg360*) | c.1078C>T (p.Arg360*) | 1 | Vitoria (2013) |
| c.1141delG (p.Val381Cysfs*27) | IVS16ins3kb | 1 | Wang (2023) |
| c.1177+1G>A (p.Val340_Arg392del) | c.1177+1G>A (p.Val340_Arg392del) | 11 | Our study |
| c.1177+1G>A (p.Val340_Arg392del) | c.1311+1G>A (p.Val411_Cys437del) | 5 | Our study |
| c.1177+1G>A (p.Val340_Arg392del) | c.1511A>G (p.Tyr504Cys) | 1 | Our study |
| c.1177+1G>A (p.Val340_Arg392del) | c.1592G>A (p.Gly531Asp) | 1 | Our study |
| c.1177+1G>A (p.Val340_Arg392del) | c.1638_1660dup (p.Ala554Glyfs*17) | 1 | Our study |
| c.1177+1G>A (p.Val340_Arg392del) | IVS16ins3kb | 1 | Our study |
| c.1177+1G>A (p.Val340_Arg392del) | ND | 1 | Our study |
| c.1177+1G>A (p.Val340_Arg392del) | c.1813C>T (p.Arg605*) | 1 | Our study |
| c.1230+1G>A (p.?) | IVS16ins3kb | 1 | Our study |
| c.1311+1G>A (p.Val411_Cys437del) | c.1311+1G>A (p.Val411_Cys437del) | 1 | Our study |
| c.1311+1G>A (p.Val411_Cys437del) | IVS16ins3kb | 1 | Our study |
| c.1311+1G>A (p.Val411_Cys437del) | ND | 1 | Our study |
| c.1638_1660dup (p.Ala554Glyfs*17) | c.1800C>G (p.Tyr600*) | 1 | Wang (2023) |
| IVS16ins3kb | IVS16ins3kb | 2 | Zhang MH (2015),  Ngu (2010) |

IVS16ins3kb: c.1750_1751[insNM_138459.3:2672_24;1750+72_1751-4dup], ND: not detectable

J. Increased blood alpha-fetoprotein levels (N=105)

| Allele 1 | Allele 2 | # Patients | Study |
| --- | --- | --- | --- |
| c.-3251_15+ 18443del21709 | c.852_855del (p.Met285Profs*2) | 1 | Zhang ZH (2017) |
| c.15G>A (p.?) | c.1311+1G>A (p.Val411_Cys437del) | 1 | Our study |
| c.(69+1_70-1)_(212+1_231-1)del | c.852_855del (p.Met285Profs*2) | 1 | Grunert (2020) |
| c.127C>T (p.Arg43*) | c.1063C>T (p.Arg355*) | 1 | Dimmock (2009) |
| c.329-1687_c.468+3865del5692bp | IVS16ins3kb | 1 | Zheng (2016) |
| c.478del (p.Leu160Trpfs*36) | c.478del (p.Leu160Trpfs*36) | 1 | Kose (2020) |
| c.615+5G>A (p.Ala206Valfs*7) | IVS16ins3kb | 1 | Lin Y (2020) |
| c.615+5G>A (p.Ala206Valfs*7) | ND | 1 | Lee (2006) |
| c.640C>T (p.Gln214*) | c.640C>T (p.Gln214*) | 1 | Kose (2020) |
| c.640C>T (p.Gln214*) | c.1709_1710insA (p.Arg571Alafs2*) | 1 | Wang (2015) |
| c.674C>A (p.Ser225*) | c.674C>A (p.Ser225*) | 1 | Tamamori (2002) |
| c.674C>A (p.Ser225*) | c.852_855del (p.Met285Profs*2) | 1 | Ko (2007) |
| c.674C>A (p.Ser225*) | c.1177+1G>A (p.Val340_Arg392del) | 5 | Our study |
| c.674C>A (p.Ser225*) | c.1638_1660dup (p.Ala554Glyfs*17) | 2 | Our study,  Ko (2007) |
| c.674C>A (p.Ser225*) | IVS16ins3kb | 1 | Our study |
| c.674C>A (p.Ser225*) | c.1799dupA (p.Tyr600*) | 1 | Our study |
| c.686delA (p.Asn229Thrfs*21) | c.686delA (p.Asn229Thrfs*21) | 2 | Tomé (2021) |
| c.848G>T (p.G283V) | c.848G>T (p.G283V) | 1 | Tal (2019) |
| c.852_855del (p.Met285Profs*2) | c.852_855del (p.Met285Profs*2) | 5 | Our study,  Lee (2006) |
| c.852_855del (p.Met285Profs*2) | c.1078C>T (p.Arg360*) | 1 | Our study |
| c.852_855del (p.Met285Profs*2) | c.1177+1G>A (p.Val340_Arg392del) | 12 | Our study,  Wang (2023),  Tamamori (2002) |
| c.852_855del (p.Met285Profs*2) | c.1230+1G>A (p.?) | 1 | Our study |
| c.852_855del (p.Met285Profs*2) | c.1638_1660dup (p.Ala554Glyfs*17) | 1 | Our study |
| c.852_855del (p.Met285Profs*2) | IVS16ins3kb | 2 | Our study,  Takeuchi (2015) |
| c.852_855del (p.Met285Profs*2) | c.1801G>T (p.Glu601*) | 1 | Our study |
| c.852_855del (p.Met285Profs*2) | c.1841+3_1841+4del (p.?) | 1 | Zhang L (2019) |
| c.852_855del (p.Met285Profs*2) | ND | 8 | Our study,  Chew (2010),  Tamamori (2002) |
| c.1018+1G>A (p.?) | c.1638_1660dup (p.Ala554Glyfs*17) | 1 | Our study |
| c.1078C>T (p.Arg360*) | c.1078C>T (p.Arg360*) | 1 | Vitoria (2013) |
| c.1141delG (p.Val381Cysfs*27) | IVS16ins3kb | 1 | Wang (2023) |
| c.1177+1G>A (p.Val340_Arg392del) | c.1177+1G>A (p.Val340_Arg392del) | 11 | Our study |
| c.1177+1G>A (p.Val340_Arg392del) | c.1311+1G>A (p.Val411_Cys437del) | 7 | Our study |
| c.1177+1G>A (p.Val340_Arg392del) | c.1511A>G (p.Tyr504Cys) | 1 | Our study |
| c.1177+1G>A (p.Val340_Arg392del) | IVS16ins3kb | 3 | Our study, Hayasaka (2012) |
| c.1177+1G>A (p.Val340_Arg392del) | c.1801G>T (p.Glu601*) | 4 | Our study,  Havasaka (2012),  Tamamori (2002) |
| c.1177+1G>A (p.Val340_Arg392del) | c.1813C>T (p.Arg605*) | 1 | Our study |
| c.1177+1G>A (p.Val340_Arg392del) | ND | 3 | Our study |
| c.1230+1G>A (p.?) | IVS16ins3kb | 1 | Our study |
| c.1307_1308delinsAA (p.Gly436Glu) | c.1307_1308delinsAA (p.Gly436Glu) | 1 | Fiermonte (2011) |
| c.1311+1G>A (p.Val411_Cys437del) | c.1311+1G>A (p.Val411_Cys437del) | 3 | Our study |
| c.1311+1G>A (p.Val411_Cys437del) | IVS16ins3kb | 1 | Our study |
| c.1311+1G>A (p.Val411_Cys437del) | c.1801G>T (p.Glu601*) | 1 | Our study |
| c.1354G>A (p.Val452Ile) | c.1354G>A (p.Val452Ile) | 1 | Seker-Yilmaz (2017) |
| c.1453-1G>A (p.?) | IVS16ins3kb | 1 | Lin WX (2021) |
| c.1465T>C (p.Cys489Arg) | ND | 1 | Hutchin (2009) |
| c.1638_1660dup (p.Ala554Glyfs*17) | ND | 1 | Ko (2007) |
| c.1638_1660dup (p.Ala554Glyfs*17) | c.1800C>G (p.Tyr600*) | 1 | Wang (2023) |
| IVS16ins3kb | IVS16ins3kb | 4 | Chew (2010), Ngu (2010) |

IVS16ins3kb: c.1750_1751[insNM_138459.3:2672_24;1750+72_1751-4dup], ND: not detectable

K. The onset within 1 months after birth (N=132)

| Allele 1 | Allele 2 | # Patients | Study |
| --- | --- | --- | --- |
| c.46G>T (p.Glu16*) | c.852_855del (p.Met285Profs*2) | 1 | Our study |
| c.74C>A (p.A25E) | c.74C>A (p.A25E) | 1 | Dimmock (2009) |
| c.135G>C (p.L45F) | c.615+5G>A (p.A206Vfs*7) | 1 | Sun (2023) |
| c.173_174delTG (p.V58Gfs*24) | c.173_174delTG (p.V58Gfs*24) | 1 | Dimmock (2009) |
| c.615+1G>C (p.Ala206Leufs*7) | c.852_855del (p.Met285Profs*2) | 1 | Dimmock (2009) |
| c.615+1G>C (p.Ala206Leufs*7) | ND | 1 | Our study |
| c.674C>A (p.Ser225*) | c.674C>A (p.Ser225*) | 1 | Tamamori (2002) |
| c.674C>A (p.Ser225*) | c.852_855del (p.Met285Profs*2) | 1 | Our study |
| c.674C>A (p.Ser225*) | c.1177+1G>A (p.Val340_Arg392del) | 6 | Our study |
| c.674C>A (p.Ser225*) | c.1638_1660dup (p.Ala554Glyfs*17) | 1 | Our study |
| c.674C>A (p.Ser225*) | c.1799dupA (p.Tyr600*) | 2 | Our study |
| c.674C>A (p.Ser225*) | ND | 2 | Our study |
| c.847G>T (p.G283*) | c.852_855del (p.Met285Profs*2) | 1 | Our study |
| c.848+3A>C | c.1637C>G (p.T546R) | 1 | Dimmock (2009) |
| c.848+6T>C | c.848+6T>C | 1 | Sachs (2023) |
| c.852_855del (p.Met285Profs*2) | c.852_855del (p.Met285Profs*2) | 6 | Our study |
| c.852_855del (p.Met285Profs*2) | c.1018+1G>A (p.?) | 1 | Our study |
| c.852_855del (p.Met285Profs*2) | c.1078C>T (p.Arg360*) | 2 | Our study |
| c.852_855del (p.Met285Profs*2) | c.1177+1G>A (p.Val340_Arg392del) | 22 | Our study |
| c.852_855del (p.Met285Profs*2) | c.1311+1G>A (p.Val411_Cys437del) | 3 | Our study |
| c.852_855del (p.Met285Profs*2) | c.1638_1660dup (p.Ala554Glyfs*17) | 1 | Our study |
| c.852_855del (p.Met285Profs*2) | IVS16ins3kb | 4 | Our study |
| c.852_855del (p.Met285Profs*2) | c.1793 T>G (p.Leu598Arg) | 1 | Our study |
| c.852_855del (p.Met285Profs*2) | c.1799dupA (p.Tyr600*) | 2 | Our study |
| c.852_855del (p.Met285Profs*2) | c.1801G>T (p.Glu601*) | 1 | Our study |
| c.852_855del (p.Met285Profs*2) | ND | 9 | Our study  Pinto (2020)  Chew (2010)  Tamamori (2002) |
| c.1018+1G>A (p.?) | c.1018+1G>A (p.?) | 2 | Our study |
| c.1018+1G>A (p.?) | c.1638_1660dup (p.Ala554Glyfs*17) | 1 | Our study |
| c.1078C>T (p.R360*) | c.1078C>T (p.R360*) | 1 | Vitoria (2013) |
| c.1177+1G>A (p.Val340_Arg392del) | c.1177+1G>A (p.Val340_Arg392del) | 20 | Our study |
| c.1177+1G>A (p.Val340_Arg392del) | c.1311+1G>A (p.Val411_Cys437del) | 6 | Our study |
| c.1177+1G>A (p.Val340_Arg392del) | c.1511A>G (p.Tyr504Cys) | 1 | Our study |
| c.1177+1G>A (p.Val340_Arg392del) | c.1592G>A (p.Gly531Asp) | 1 | Our study |
| c.1177+1G>A (p.Val340_Arg392del) | c.1638_1660dup (p.Ala554Glyfs*17) | 2 | Our study |
| c.1177+1G>A (p.Val340_Arg392del) | IVS16ins3kb | 3 | Our study |
| c.1177+1G>A (p.Val340_Arg392del) | c.1801G>T (p.Glu601*) | 2 | Our study |
| c.1177+1G>A (p.Val340_Arg392del) | c.1813C>T (p.Arg605*) | 1 | Our study |
| c.1177+1G>A (p.Val340_Arg392del) | ND | 2 | Our study |
| c.1216dupG (p.A406Gfs*13) | IVS16ins3kb | 1 | Sun (2023) |
| c.1230+1G>A | IVS16ins3kb | 1 | Our study |
| c.1311+1G>A (p.Val411_Cys437del) | c.1311+1G>A (p.Val411_Cys437del) | 2 | Our study |
| c.1311+1G>A (p.Val411_Cys437del) | c.1801G>T (p.Glu601*) | 1 | Our study |
| c.1311+1G>A (p.Val411_Cys437del) | IVS16ins3kb | 1 | Our study |
| c.1311+1G>A (p.Val411_Cys437del) | ND | 1 | Our study |
| c.1638_1660dup (p.Ala554Glyfs*17) | c.1638_1660dup (p.Ala554Glyfs*17) | 1 | Dimmock (2009) |
| c.1638_1660dup (p.Ala554Glyfs*17) | ND | 1 | Our study |
| IVS16ins3kb | ND | 1 | Chew (2010) |
| c.1763G>A (p.R588Q) | ND | 6 | Pinto (2020) |

IVS16ins3kb: c.1750_1751[insNM_138459.3:2672_24;1750+72_1751-4dup], ND: not detectable

L. Lowered birth weight (< 2,500g) (N=93)

| Allele 1 | Allele 2 | # Patients | Study |
| --- | --- | --- | --- |
| c.2T>C (p.Met1Thr) | c.790G>A (p.Val264Ile) | 1 | Zeng (2014) |
| c.46G>T (p.Glu16*) | c.852_855del (p.Met285Profs*2) | 1 | Our study |
| c.127C>T (p.Arg43*) | c.1063C>T (p.Arg355*) | 1 | Dimmock (2009) |
| c.674C>A (p.Ser225*) | c.674C>A (p.Ser225*) | 1 | Tamamori (2002) |
| c.674C>A (p.Ser225*) | c.852_855del (p.Met285Profs*2) | 2 | Our study |
| c.674C>A (p.Ser225*) | c.1177+1G>A (p.Val340_Arg392del) | 4 | Our study |
| c.674C>A (p.Ser225*) | IVS16ins3kb | 1 | Our study |
| c.674C>A (p.Ser225*) | c.1799dup (p.Tyr600*) | 1 | Our study |
| c.674C>A (p.Ser225*) | ND | 1 | Our study |
| c.686del (p.Asn229Thrfs*21) | c.686del (p.Asn229Thrfs*21) | 1 | Tomé (2021) |
| c.754G>A (p.Glu252Lys) | c.1177+1G>A (p.Val340_Arg392del) | 1 | Lin WX (2012) |
| c.848+3A>C (p.?) | c.848+3A>C (p.?) | 1 | Tal (2019) |
| c.848+3A>C (p.?) | c.1637C>G (p.Thr546Arg) | 1 | Dimmock (2009) |
| c.852_855del (p.Met285Profs*2) | c.852_855del (p.Met285Profs*2) | 7 | Our study,  Sun (2023),  Chew (2010) |
| c.852_855del (p.Met285Profs*2) | c.1018+1G>A (p.?) | 1 | Our study |
| c.852_855del (p.Met285Profs*2) | c.1177+1G>A (p.Val340_Arg392del) | 17 | Our study,  Wang (2023),  Zeng (2016),  Tamamori (2002) |
| c.852_855del (p.Met285Profs*2) | c.1311+1G>A (p.Val411_Cys437del) | 4 | Our study |
| c.852_855del (p.Met285Profs*2) | c.1638_1660dup (p.Ala554Glyfs*17) | 2 | Our study |
| c.852_855del (p.Met285Profs*2) | IVS16ins3kb | 4 | Our study |
| c.852_855del (p.Met285Profs*2) | ND | 6 | Tamamori (2002),  Our study |
| c.1018+1G>A (p.?) | c.1018+1G>A (p.?) | 1 | Our study |
| c.1177+1G>A (p.Val340_Arg392del) | c.1177+1G>A (p.Val340_Arg392del) | 11 | Our study |
| c.1177+1G>A (p.Val340_Arg392del) | c.1311+1G>A (p.Val411_Cys437del) | 5 | Our study |
| c.1177+1G>A (p.Val340_Arg392del) | c.1638_1660dup (p.Ala554Glyfs*17) | 2 | Our study |
| c.1177+1G>A (p.Val340_Arg392del) | IVS16ins3kb | 3 | Our study |
| c.1177+1G>A (p.Val340_Arg392del) | ND | 3 | Our study,  Hayasaka (2012) |
| c.1177+1G>A (p.Val340_Arg392del) | c.1813C>T (p.Arg605*) | 1 | Our study |
| c.1230+1G>A (p.?) | IVS16ins3kb | 2 | Our study |
| c.1311+1G>A (p.Val411_Cys437del) | IVS16ins3kb | 1 | Our study |
| c.1665_1842-32del516 (p.?) | c.1665_1842-32del516 (p.?) | 1 | Takaya (2005) |
| IVS16ins3kb | IVS16ins3kb | 4 | Chew (2010), Ngu (2010) |
| IVS16ins3kb | ND | 1 | Chew (2010) |

IVS16ins3kb: c.1750_1751[insNM_138459.3:2672_24;1750+72_1751-4dup], ND: not detectable

Supplemental data 3. Low-frequent clinical manifestations and variants distribution in the 285 patients with NICCD and 19 patients with post-NICCD

A. Kidney injury (N=4)

| Allele 1 | Allele 2 | #Patients | Study |
| --- | --- | --- | --- |
| c.852_855del (p.Met285Profs*2) | c.1793T>G (p.Leu598Arg) | 1 | Our study |
| c.1018+1G>A (p.?) | c.1018+1G>A (p.?) | 2 | Our study |
| c.1311+1G>A (p.Val411_Cys437del) | IVS16ins3kb | 1 | Our study |

IVS16ins3kb: IVS16ins3kb: c.1750_1751[insNM_138459.3:2672_24;1750+72_1751-4dup]

B. Thrombocytopenia (Plt < 10×10^4^/μL) (N=4)

| Allele 1 | Allele 2 | #Patients | Study |
| --- | --- | --- | --- |
| c.852_855del (p.Met285Profs*2) | c.852_855del (p.Met285Profs*2) | 1 | Our study |
| c.852_855del (p.Met285Profs*2) | c.1311+1G>A (p.Val411_Cys437del) | 1 | Our study |
| c.852_855del (p.Met285Profs*2) | c.1793T>G (p.Leu598Arg) | 1 | Our study |
| c.1177+1G>A (p.Val340_Arg392del) | c.1311+1G>A (p.Val411_Cys437del) | 1 | Our study |

C. Bleeding tendency (N=6)

| Allele 1 | Allele 2 | #Patients | Study |
| --- | --- | --- | --- |
| c.674C>A (p.Ser225*) | c.852_855del (p.Met285Profs*2) | 1 | Our study |
| c.674C>A (p.Ser225*) | c.1799dup (p.Tyr600*) | 1 | Our study |
| c.852_855del (p.Met285Profs*2) | c.1311+1G>A (p.Val411_Cys437del) | 1 | Our study |
| c.1177+1G>A (p.Val340_Arg392del) | c.1177+1G>A (p.Val340_Arg392del) | 2 | Our study |
| c.1177+1G>A (p.Val340_Arg392del) | c.1311+1G>A (p.Val411_Cys437del) | 1 | Our study |

D. Intellectual disability (N=5)

| Allele 1 | Allele 2 | #Patients | Study |
| --- | --- | --- | --- |
| c.615+1G>C (p.Ala206Leufs*7) | ND | 1 | Our study |
| c.674C>A (p.Ser225*) | c.1177+1G>A (p.Val340_Arg392del) | 1 | Our study |
| c.1177+1G>A (p.Val340_Arg392del) | c.1311+1G>A (p.Val411_Cys437del) | 1 | Our study |
| c.1177+1G>A (p.Val340_Arg392del) | ND | 1 | Our study |
| c.1311+1G>A (p.Val411_Cys437del) | c.1311+1G>A (p.Val411_Cys437del) | 1 | Our study |

ND: not detectable

E. Autism (N=3)

| Allele 1 | Allele 2 | #Patients | Study |
| --- | --- | --- | --- |
| c.1177+1G>A (p.Val340_Arg392del) | c.1311+1G>A (p.Val411_Cys437del) | 1 | Our study |
| c.1177+1G>A (p.Val340_Arg392del) | c.1638_1660dup (p.Ala554Glyfs*17) | 1 | Our study |
| c.1177+1G>A (p.Val340_Arg392del) | ND | 1 | Our study |

ND: not detectable

F. Liver transplant (N=3)

| Allele 1 | Allele 2 | Patients | Study |
| --- | --- | --- | --- |
| c.686del (p.Asn229Thrfs*21) | c.686del (p.Asn229Thrfs*21) | 2 | Tomé (2021) |
| c.852_855del (p.Met285Profs*2) | c.1177+1G>A (p.Val340_Arg392del) | 1 | Tamamori (2002) |

Supplemental data 4. The relation of non-presentation of symptoms and variants in the 285 patients with NICCD

A. Non-cholestasis (N=24)

| Allele 1 | Allele 2 | # Patients | Study |
| --- | --- | --- | --- |
| c.674C>A (p.Ser225*) | c.1177+1G>A (p.Val340_Arg392del) | 1 | Our study |
| c.674C>A (p.Ser225*) | ND | 2 | Our study |
| c.852_855del (p.Met285Profs*2) | c.852_855del (p.Met285Profs*2) | 1 | Our study |
| c.852_855del (p.Met285Profs*2) | c.1177+1G>A (p.Val340_Arg392del) | 3 | Our study |
| c.852_855del (p.Met285Profs*2) | c.1311+1G>A (p.Val411_Cys437del) | 2 | Our study |
| c.852_855del (p.Met285Profs*2) | c.1638_1660dup (p.Ala554Glyfs*17) | 1 | Our study |
| c.852_855del (p.Met285Profs*2) | c.1793 T>G (p.Leu598Arg) | 1 | Our study |
| c.955C>T (p.Arg319*) | c.1177+1G>A (p.Val340_Arg392del) | 1 | Our study |
| c.1177+1G>A (p.Val340_Arg392del) | c.1177+1G>A (p.Val340_Arg392del) | 3 | Our study |
| c.1177+1G>A (p.Val340_Arg392del) | c.1311+1G>A (p.Val411_Cys437del) | 3 | Our study |
| c.1177+1G>A (p.Val340_Arg392del) | c.1592G>A (p.Gly531Asp) | 1 | Our study |
| c.1177+1G>A (p.Val340_Arg392del) | c.1638_1660dup (p.Ala554Glyfs*17) | 1 | Our study |
| c.1177+1G>A (p.Val340_Arg392del) | IVS16ins3kb | 2 | Our study |
| c.1177+1G>A (p.Val340_Arg392del) | c.1813C>T (p.Arg605*) | 1 | Wada (2020)  Our study |
| c.1230+1G>A (p.?) | IVS16ins3kb | 1 | Our study |

IVS16ins3kb: c.1750_1751[insNM_138459.3:2672_24;1750+72_1751-4dup], ND: not detectable

B. Non-elevated transaminase (N=40)

| Allele 1 | Allele 2 | # Patients | Study |
| --- | --- | --- | --- |
| c.615+1G>C (p.Ala206Leufs*7) | ND | 1 | Our study |
| c.674C>A (p.Ser225*) | c.852_855del (p.Met285Profs*2) | 2 | Our study |
| c.674C>A (p.Ser225*) | c.1177+1G>A (p.Val340_Arg392del) | 2 | Our study |
| c.674C>A (p.Ser225*) | c.1799dupA (p.Tyr600*) | 1 | Our study |
| c.674C>A (p.Ser225*) | ND | 2 | Our study |
| c.852_855del (p.Met285Profs*2) | c.852_855del (p.Met285Profs*2) | 2 | Our study |
| c.852_855del (p.Met285Profs*2) | c.1018+1G>A (p.?) | 1 | Our study |
| c.852_855del (p.Met285Profs*2) | c.1177+1G>A (p.Val340_Arg392del) | 6 | Our study |
| c.852_855del (p.Met285Profs*2) | c.1078C>T (p.Arg360*) | 1 | Our study |
| c.852_855del (p.Met285Profs*2) | c.1311+1G>A (p.Val411_Cys437del) | 1 | Our study |
| c.852_855del (p.Met285Profs*2) | c.1801 G>T (p.Glu601*) | 1 | Our study |
| c.955C>T (p.Arg319*) | c.1177+1G>A (p.Val340_Arg392del) | 1 | Our study |
| c.1018+1G>A (p.?) | c.1018+1G>A (p.?) | 2 | Our study |
| c.1177+1G>A (p.Val340_Arg392del) | c.1177+1G>A (p.Val340_Arg392del) | 4 | Our study |
| c.1177+1G>A (p.Val340_Arg392del) | c.1311+1G>A (p.Val411_Cys437del) | 3 | Our study |
| c.1177+1G>A (p.Val340_Arg392del) | c.1592G>A (p.Gly531Asp) | 1 | Our study |
| c.1177+1G>A (p.Val340_Arg392del) | c.1638_1660dup (p.Ala554Glyfs*17) | 1 | Our study |
| c.1177+1G>A (p.Val340_Arg392del) | IVS16ins3kb | 3 | Our study |
| c.1177+1G>A (p.Val340_Arg392del) | c.1813C>T (p.Arg605*) | 1 | Our study  Wada (2020) |
| c.1177+1G>A (p.Val340_Arg392del) | ND | 3 | Our study |
| c.1230+1G>A (p.?) | IVS16ins3kb | 1 | Our study |

IVS16ins3kb: c.1750_1751[insNM_138459.3:2672_24;1750+72_1751-4dup], ND: not detectable

C. Non-fatty liver (N=74)

| Allele 1 | Allele 2 | # Patients | Study |
| --- | --- | --- | --- |
| c.15G>A (p.?) | c.1177+1G>A (p.Val340_Arg392del) | 1 | Our study |
| c.46G>T (p.Glu16*) | c.852_855del (p.Met285Profs*2) | 1 | Our study |
| c.615+1G>C (p.Ala206Leufs*7) | ND | 1 | Our study |
| c.674C>A (p.Ser225*) | c.852_855del (p.Met285Profs*2) | 2 | Our study |
| c.674C>A (p.Ser225*) | c.1177+1G>A (p.Val340_Arg392del) | 5 | Our study |
| c.674C>A (p.Ser225*) | c.1638_1660dup (p.Ala554Glyfs*17) | 1 | Our study |
| c.674C>A (p.Ser225*) | IVS16ins3kb | 1 | Our study |
| c.674C>A (p.Ser225*) | ND | 3 | Our study |
| c.847G>T (p.Gly283*) | c.852_855del (p.Met285Profs*2) | 1 | Our study |
| c.852_855del (p.Met285Profs*2) | c.852_855del (p.Met285Profs*2) | 8 | Our study |
| c.852_855del (p.Met285Profs*2) | c.1018+1G>A (p.?) | 1 | Our study |
| c.852_855del (p.Met285Profs*2) | c.1177+1G>A (p.Val340_Arg392del) | 10 | Our study |
| c.852_855del (p.Met285Profs*2) | c.1078C>T (p.Arg360*) | 1 | Our study |
| c.852_855del (p.Met285Profs*2) | c.1311+1G>A (p.Val411_Cys437del) | 4 | Our study |
| c.852_855del (p.Met285Profs*2) | c.1638_1660dup (p.Ala554Glyfs*17) | 2 | Our study |
| c.852_855del (p.Met285Profs*2) | c.1793 T>G (p.Leu598Arg) | 1 | Our study |
| c.852_855del (p.Met285Profs*2) | c.1799dupA (p.Tyr600*) | 1 | Our study |
| c.852_855del (p.Met285Profs*2) | c.1801G>T (p.Glu601*) | 1 | Our study |
| c.852_855del (p.Met285Profs*2) | ND | 2 | Our study |
| c.1018+1G>A (p.?) | c.1018+1G>A (p.?) | 3 | Our study |
| c.1018+1G>A (p.?) | c.1638_1660dup (p.Ala554Glyfs*17) | 1 | Our study |
| c.1177+1G>A (p.Val340_Arg392del) | c.1177+1G>A (p.Val340_Arg392del) | 12 | Our study |
| c.1177+1G>A (p.Val340_Arg392del) | c.1311+1G>A (p.Val411_Cys437del) | 2 | Our study |
| c.1177+1G>A (p.Val340_Arg392del) | c.1592G>A (p.Gly531Asp) | 1 | Our study |
| c.1177+1G>A (p.Val340_Arg392del) | c.1638_1660dup (p.Ala554Glyfs*17) | 1 | Our study |
| c.1177+1G>A (p.Val340_Arg392del) | IVS16ins3kb | 3 | Our study |
| c.1177+1G>A (p.Val340_Arg392del) | c.1813C>T (p.Arg605*) | 1 | Our study, Wada (2020) |
| c.1177+1G>A (p.Val340_Arg392del) | ND | 1 | Our study |
| c.1230+1G>A (p.?) | IVS16ins3kb | 1 | Our study |
| c.1311+1G>A (p.Val411_Cys437del) | ND | 1 | Our study |

IVS16ins3kb: c.1750_1751[insNM_138459.3:2672_24;1750+72_1751-4dup], ND: not detectable

D. Non-prolonged prothrombin time (N=80)

| Allele 1 | Allele 2 | # Patients | Study |
| --- | --- | --- | --- |
| c.674C>A (p.Ser225*) | c.852_855del (p.Met285Profs*2) | 1 | Our study |
| c.674C>A (p.Ser225*) | c.1177+1G>A (p.Val340_Arg392del) | 6 | Our study |
| c.674C>A (p.Ser225*) | c.1799dupA (p.Tyr600*) | 1 | Our study |
| c.852_855del (p.Met285Profs*2) | c.852_855del (p.Met285Profs*2) | 7 | Our study |
| c.852_855del (p.Met285Profs*2) | c.1018+1G>A (p.?) | 1 | Our study |
| c.852_855del (p.Met285Profs*2) | c.1177+1G>A (p.Val340_Arg392del) | 11 | Our study |
| c.852_855del (p.Met285Profs*2) | c.1078C>T (p.Arg360*) | 2 | Our study |
| c.852_855del (p.Met285Profs*2) | c.1230+1G>A (p.?) | 1 | Our study |
| c.852_855del (p.Met285Profs*2) | c.1311+1G>A (p.Val411_Cys437del) | 5 | Our study |
| c.852_855del (p.Met285Profs*2) | c.1638_1660dup (p.Ala554Glyfs*17) | 1 | Our study |
| c.852_855del (p.Met285Profs*2) | IVS16ins3kb | 2 | Our study |
| c.852_855del (p.Met285Profs*2) | c.1799dupA (p.Tyr600*) | 1 | Our study |
| c.852_855del (p.Met285Profs*2) | c.1801 G>T (p.Glu601*) | 1 | Our study |
| c.852_855del (p.Met285Profs*2) | c.1177+1G>A (p.Val340_Arg392del) | 1 | Wang (2023) |
| c.852_855del (p.Met285Profs*2) | ND | 2 | Our study |
| c.1018+1G>A (p.?) | c.1638_1660dup (p.Ala554Glyfs*17) | 1 | Our study |
| c.1177+1G>A (p.Val340_Arg392del) | c.1177+1G>A (p.Val340_Arg392del) | 15 | Our study |
| c.1177+1G>A (p.Val340_Arg392del) | c.1311+1G>A (p.Val411_Cys437del) | 5 | Our study |
| c.1177+1G>A (p.Val340_Arg392del) | c.1592G>A (p.Gly531Asp) | 2 | Our study |
| c.1177+1G>A (p.Val340_Arg392del) | c.1638_1660dup (p.Ala554Glyfs*17) | 3 | Our study |
| c.1177+1G>A (p.Val340_Arg392del) | IVS16ins3kb | 2 | Our study |
| c.1177+1G>A (p.Val340_Arg392del) | c.1801G>T (p.Glu601*) | 2 | Our study |
| c.1177+1G>A (p.Val340_Arg392del) | c.1813C>T (p.Arg605*) | 1 | Our study,  Wada (2020) |
| c.1230+1G>A (p.?) | IVS16ins3kb | 1 | Our study |
| c.1311+1G>A (p.Val411_Cys437del) | c.1311+1G>A (p.Val411_Cys437del) | 2 | Our study |
| c.1311+1G>A (p.Val411_Cys437del) | IVS16ins3kb | 1 | Our study |
| c.1311+1G>A (p.Val411_Cys437del) | c.1801G>T (p.Glu601*) | 1 | Our study |
| IVS16ins3kb | c.1800C>G (p.Tyr600*) | 1 | Wang (2023) |

IVS16ins3kb: c.1750_1751[insNM_138459.3:2672_24;1750+72_72_1751-4dup], ND: not detectable

Supplemental data 5. Clinical manifestations and variants distribution in the 41 patients with AACD.

A. Hyperammonemia (≥ 180 μmol/L) (N=29)

| Allele 1 | Allele 2 | #Patients | Study |
| --- | --- | --- | --- |
| c.650del (p.Phe217Serfs*33) | c.869T>C (p.Ile290Thr) | 1 | Bijarnia-Mahay (2015) |
| c.674C>A (p.Ser225*) | c.1177+1G>A (p.Val340_Arg392del) | 1 | Tazawa (2013) |
| c.674C>A (p.Ser225*) | c.1311+1G>A (p.Val411_Cys437del) | 1 | Hayasaka (2014) |
| c.674C>A (p.Ser225*) | c.1478A>G (p.Asp493Gly) | 2 | Our study, Takahashi (2012) |
| c.674C>A (p.Ser225*) | c.1645C>T (p.Glu549*) | 2 | Our study,  Hayasaka (2014) |
| c.852_855del (p.M285Pfs*2) | c.852_855del (p.M285Pfs*2) | 5 | Our study,  Unita (2020), Hayasaka (2018), Tang (2016), Tsai (2006) |
| c.852_855del (p.M285Pfs*2) | c.1070A>G (p.Gln357Arg) | 1 | Yazaki (2012) |
| c.852_855del (p.M285Pfs*2) | c.1177+1G>A (p.Val340_Arg392del) | 4 | Our study  Hayasaka (2018),  Yazaki (2012) |
| c.852_855del (p.M285Pfs*2) | c.1231G>A (p.Val411Met) | 1 | Ng (2011) |
| c.852_855del (p.M285Pfs*2) | c.1311+1G>A (p.Val411_Cys437del) | 1 | Fukushima (2010) |
| c.852_855del (p.M285Pfs*2) | IVS16ins3kb | 1 | Our study |
| c.852_855del (p.M285Pfs*2) | c.1813C>T (p.Arg605*) | 2 | Our study,  Hayasaka (2014) |
| c.1177+1G>A (p.Val340_Arg392del) | c.1177+1G>A (p.Val340_Arg392del) | 2 | Our study,  Hayasaka (2018) |
| c.1177+1G>A (p.Val340_Arg392del) | c.1801G>T (p.Glu601*) | 1 | Hayasaka (2018) |
| c.1311+1G>A (p.Val411_Cys437del) | IVS16ins3kb | 1 | Our study |
| c.1311+1G>A (p.Val411_Cys437del) | c.1801G>T (p.Glu601*) | 1 | Our study |
| c.1478A>G (p.Asp493Gly) | c.1478A>G (p.Asp493Gly) | 1 | Kose (2020) |
| c.1592G>A (p.Gly531Asp) | c.1592G>A (p.Gly531Asp) | 1 | Our study |

IVS16ins3kb: c.1750_1751[insNM_138459.3:2672_24;1750+72_1751-4dup]

B. Impaired consciousness (N=10)

| Allele 1 | Allele 2 | #Patients | Study |
| --- | --- | --- | --- |
| c.674C>A (p.Ser225*) | c.1478A>G (p.Asp493Gly) | 1 | Our study |
| c.674C>A (p.Ser225*) | c.1645C>T (p.Glu549*) | 1 | Our study |
| c.852_855del (p.Met285Profs*2) | c.852_855del (p.Met285Profs*2) | 1 | Our study |
| c.852_855del (p.Met285Profs*2) | c.1177+1G>A (p.Val340_Arg392del) | 2 | Our study |
| c.852_855del (p.Met285Profs*2) | IVS16ins3kb | 1 | Our study |
| c.852_855del (p.Met285Profs*2) | c.1813C>T (p.Arg605*) | 1 | Our study |
| c.1311+1G>A (p.Val411_Cys437del) | IVS16ins3kb | 1 | Our study |
| c.1311+1G>A (p.Val411_Cys437del) | c.1801G>T (p.Glu601*) | 1 | Our study |
| c.1592G>A (p.Gly531Asp) | c.1592G>A (p.Gly531Asp) | 1 | Our study |

IVS16ins3kb: c.1750_1751[insNM_138459.3:2672_24;1750+72_1751-4dup]

C. Intellectual disability (N=4)

| Allele 1 | Allele 2 | #Patients | Study |
| --- | --- | --- | --- |
| c.674C>A (p.Ser225*) | c.1645C>T (p.Glu549*) | 1 | Our study |
| c.852_855del (p.Met285Profs*2) | c.852_855del (p.Met285Profs*2) | 1 | Our study |
| c.1018+1G>A (p.?) | c.1018+1G>A (p.?) | 1 | Our study |
| c.1177+1G>A (p.Val340_Arg392del) | c.1177+1G>A (p.Val340_Arg392del) | 1 | Our study |

D. Short stature (in the adult age) (N=6)

| Allele 1 | Allele 2 | #Patients | Study |
| --- | --- | --- | --- |
| c.852_855del (p.Met285Profs*2) | c.852_855del (p.Met285Profs*2) | 1 | Our study |
| c.852_855del (p.Met285Profs*2) | c.1177+1G>A (p.Val340_Arg392del) | 1 | Our study |
| c.852_855del (p.Met285Profs*2) | IVS16ins3kb | 1 | Our study |
| c.1018+1G>A (p.?) | c.1018+1G>A (p.?) | 1 | Our study |
| c.1311+1G>A (p.Val411_Cys437del) | IVS16ins3kb | 1 | Our study |
| c.1592G>A (p.Gly531Asp) | c.1592G>A (p.Gly531Asp) | 1 | Our study |

IVS16ins3kb: c.1750_1751[insNM_138459.3:2672_24;1750+72_1751-4dup]

E. Liver cirrhosis (N=3)

| Allele 1 | Allele 2 | #Patients | Study |
| --- | --- | --- | --- |
| c.852_855del (p.Met285Profs*2) | c.852_855del (p.Met285Profs*2) | 1 | Our study |
| c.1018+1G>A (p.?) | c.1018+1G>A (p.?) | 1 | Our study |
| c.1311+1G>A (p.Val411_Cys437del) | IVS16ins3kb | 1 | Our study |

IVS16ins3kb: c.1750_1751[insNM_138459.3:2672_24;1750+72_1751-4dup]

F. Pancreatitis (N=4)

| Allele 1 | Allele 2 | #Patients | Study |
| --- | --- | --- | --- |
| c.852_855del (p.Met285Profs*2) | c.852_855del (p.Met285Profs*2) | 1 | Our study |
| c.852_855del (p.Met285Profs*2) | IVS16ins3kb | 1 | Our study |
| c.1311+1G>A (p.Val411_Cys437del) | IVS16ins3kb | 1 | Our study |
| c.1311+1G>A (p.Val411_Cys437del) | c.1801G>T (p.Glu601*) | 1 | Our study |

IVS16ins3kb: c.1750_1751[insNM_138459.3:2672_24;1750+72_1751-4dup]

G. Liver transplantation (N=7)

| Allele 1 | Allele 2 | Patients | Study |
| --- | --- | --- | --- |
| c.674C>A (p.Ser225*) | c.1177+1G>A (p.Val340_Arg392del) | 1 | Tazawa (2013) |
| c.852_855del (p.Met285Profs*2) | c.1070A>G (p.Gln357Arg) | 1 | Yazaki (2012) |
| c.852_855del (p.Met285Profs*2) | c.1177+1G>A (p.Val340_Arg392del) | 1 | Yazaki (2012) |
| c.852_855del (p.Met285Profs*2) | c.1311+1G>A (p.Val411_Cys437del) | 1 | Kogure (2014) |
| c.852_855del (p.Met285Profs*2) | IVS16ins3kb | 1 | Chen (2021) |
| c.1018+1G>A (p.?) | c.1018+1G>A (p.?) | 1 | Our study |
| c.1177+1G>A (p.Val340_Arg392del) | c.1177+1G>A (p.Val340_Arg392del) | 1 | Hayasaka (2018) |

IVS16ins3kb: c.1750_1751[insNM_138459.3:2672_24;1750+72_1751-4dup]
